# Experimental evaluation of the effect of positioning and operating parameters on the performance of a surface-piercing propeller

**DOI:** 10.1038/s41598-022-21959-x

**Published:** 2022-11-03

**Authors:** Maryam Kamran, Norouz Mohammad Nouri, Hossein Goudarzi, Saeed Golrokhifar

**Affiliations:** School of Mechanical Engineering, Iran University of Science and Technology, Tehran, Iran

**Keywords:** Mechanical engineering, Fluid dynamics, Characterization and analytical techniques

## Abstract

Today, surface-piercing propellers have been recognized as a suitable choice for higher speeds. Yet, the development of design algorithms for such has been challenged by insufficient knowledge about the parameters affecting their performance. For this reason, developing experimental data and studying the influence of various parameters on their performance is crucial. Aiming to develop experimental knowledge of these propellers, this study investigates the impact of position parameters and Froude Number on model test results of a custom-designed propeller. Moreover, ventilation wake development at different Froude numbers was studied. The experimental results pointed to the favorable impact of increased immersion ratio on propeller's thrust, a positive impact of increasing the inclination angle by 6° on higher thrust and efficiency in the advance direction, and a slight increase of thrust with higher yaw angles up to 10°. The propeller's lateral forces were also extracted in different positions and operational conditions to identify the propeller's behavior and design the required shaft and supports. Finally, regression equations for projecting hydrodynamic coefficients used at the design phase were compared and verified by the experimental results. The results pointed to the insufficient precision of this model for estimating the hydrodynamic coefficients affecting the propeller.

## Introduction

The notion of using surface drive systems and surface-piercing propellers (SPPs) was first initiated for shallow-draft boat propulsion^1^, as the process of increasing speed in conventional propellers results in two detrimental factors in propeller performance: (1) cavitation, which is extensively adverse, and as it cannot be ignored at high speeds, the supercavitation phenomenon on the blade suction surface is considered. This solution has prevented the negative impact of microbubbles but decreased the propeller efficiency at the same time by limiting the pressure behind the blade to the cavitation vapor pressure; and (2) at high speeds, the hydrodynamic drag force is increased on propeller protective structure and shaft, which thus lowers the system efficiency. In order to address these issues, designers of high-speed boats changed the propeller's installed position in a manner that the shaft line would be aligned with the draft line of the vessel. Here, each blade rotates at the interface between water and air, providing for the ventilation phenomenon in the blade’s backside that prevents cavitation. In this propulsion system, a part of the propeller is the only component to contact water, which drastically reduces the resistance of the system parts^2^. In this manner, the final speed and efficiency increase while decreasing fuel consumption. Further advantages of using surface-piercing propellers include higher carriage capacity per power unit, possibility to increase propeller diameter due to its distance from the stern, and flexible shaft angles, which control lift and side force results in better maneuverability.

Despite the mentioned advantages of such propulsion systems, researchers have been hindered by the complicated physics and multi-phase flow around the propellers in obtaining a complete understanding of the impact of different parameters on their performance and thus devising a standard method (similar to the ones developed for conventional propellers) to design their geometries for the intended performance. Such insufficient knowledge would result in additional costs. The published information about surface-piercing propellers only includes limited geometries, and the information is not fully accessible due to the limited field of application. Any efforts to design such propellers have entailed a trial-and-error process or followed the experimental studies conducted so far^3^.

In the attempt to identify effective parameters in the design and performance of SPPs, different experimental studies have been performed, which can be divided into the two groups of studies focusing on identifying the effective parameters in the experimental testing process and those studying the impact of different parameters on the performance of surface-piercing propellers.

The hydrodynamic data related to propeller performance is usually obtained through experimental methods and testing scaled models in water tunnels or towing tanks, while full-scale propellers are rarely tested due to economic reasons. Thus, some experimental studies have been carried out so far to determine the requirements for the SPP model test. In this regard, Shiba introduced dimensionless numbers effective on the ventilation cavity and showed that for Weber numbers above 180, propeller ventilation and critical advance ratio would behave independently from surface tension^4^. Then, Hadler and Hecker identified full and partial ventilation areas in several 3-blade SPPs^5^. They observed air ventilation forming on the blade's edge within the partial ventilation area, which increased lift to drag ratio and propeller efficiency. But under full ventilation, air cavity cover the blade's entire backside and seriously decrease the SPP efficiency. Shields demonstrated that the hydrodynamic behavior of a supercavitating propeller with Froude numbers higher than 4 is independent of this number, while lower Froude numbers increased the force on the blade^6^. Kruppa also introduced the Froude number and cavitation number as an effective parameter for generalizing model test results to full-scale propellers^7^. Brandt investigated vapor cavities in SPPs operating either fully or partially ventilated area, and identified the effect of different dimensionless numbers in sub-regimes of SPP flow pattern^8^. After that, using surface pressure changes in the cavitation tunnel, Rose et al. evaluated lateral forces and hydrodynamic coefficients of a SPP by adapting the model's cavitation number and full-scale propellers. They found that at lower immersion depths, the vertical force to thrust ratio decreased and the side force to thrust ratio increased^9^. Also, with an experimental test on three SPPs, Ferrando et al. observed the Weber number to affect the critical advance ratio and play an important role in hydrodynamic coefficients within the full ventilation area^10^. In 2007, Pustoshny et al. introduced the Reynolds, Froude, and Weber numbers independence ranges in an investigation of the performance of a 5-blade propeller in a towing tank^11^. Similarly, Ding studied the performance of 6-bladed propellers with different pitch ratios and demonstrated that at Froude numbers above 3.5, propeller behavior was independent of cavitation number^12^.

Besides determining propeller model testing requirements, other researchers attempted to identify the effect of various geometrical and positional parameters on propeller performance and find systematic relationships between them. In this regard, Hecker studied the performance of a 8-bladed SPP in different immersion depths and shaft inclination and yaw angles, and pointed to the immersion ratios as the most effective parameter in increasing propeller lift force^13^. As well, in the performance test of an 8-bladed SPP, Alder and Moor found that changing the yaw angle increased the propeller's efficiency^14^. In another study, Shaozong et al. analyzed hydrofoils' behavior with different sections^15^, and their results were similar to Hadler and Hecker^5^. In a comprehensive study, Olofsson evaluated the hydrodynamic performance of the 4-blade propeller (841b) at various shaft and yaw angles by attaching a transducer to the blade in the hub. he also studied the effects of Froude and cavitation numbers on various areas of the SPP's curve^16^. Kikuchi et al. considered the impact of shaft inclination angle in three SPPs with different pitch ratios at different advance ratios^17^. In a study conducted by Okada et al., focusing on identifying effective parameters for promoting performance in low-speed areas and reverse mode, compared the performance of three SPPs with different blade sections. Their results indicated that the blade trailing edge shape was more effective in reverse mode^18^. Based on the time-averaged and time-history forces and torques of 4- and 5-blade SPPs, Dyson concluded that blade number and skew angle affect the propeller's performance. Moreover, he developed a transient loading model for SPPs^19^. Using strain gauges connected to the surface of the blade, Nozawa and Takayama analyzed the surface stresses and the performance of four types of 3-blade propellers with different pitch ratios at different shaft angles and immersion ratios^20^.

In an attempt to define a regression equation between hydrodynamic coefficients, pitch angle and advance ratio, Ferrando et al. studied the impacts of shaft angle, immersion depth and pitch ratio on the performance of 4- and 5-blade propellers^2^. Similarly, Montazeri and Ghassemi using available experimental data established regression equations for the SPP's hydrodynamic coefficients^21^. Lorio considered the impacts of immersion depth and shaft inclination angles, as well as the shaft yaw angle on the performance of a 4-blade propeller in a towing tank, and announced both shaft angles as effective on propeller performance^22^. Compared to Ferrando's regression equations, he observed a large difference in the coefficient estimations in the high advance ratios, which can be attributed to the blade pitch ratio. Misra et al. conducted an experimental study on the performance of 4-blade propellers with different cup and trailing edge section geometries in different advance ratios.Their results pointed to the considerable effect of cup in generating propeller thrust. Additionally, they used artificial neural networks to formulate test results of propellers in different conditions^23^. In recent years, Shafaghat et al. (2019) analyzed the test results pertaining to a 5-blade propeller at different shaft inclination angles and immersion ratios and compared with hydrodynamic coefficients obtained by Ferrando and Ghasemi regression equations^24^. The results showed that the geometric and position parameters of any propeller can affect the accuracy of these equations. Therefore, it is necessary to further develop and study them for different geometries and identify the effective parameters. Lastly, Amini et al. studied the effect of forced aeration behind a SPP^25^. They found that the propeller's enhanced performance decreased with an increasing immersion ratio, while the area related to enhanced performance was drawn to higher advance coefficients.

The costly and challenging nature of full-scale propeller tests in real conditions to verify SPP designs, assess their performance and study the effective parameters when installing the propeller on the vessel further highlight the importance and necessity of model testing propellers under position parameters. The present study investigated the hydrodynamic behavior of a custom-designed 4-blade surface-piercing propeller with a special blade section. This propeller was designed with a diameter of 60 cm to create a speed of 40 knots and a thrust of 12 kN, based on available experimental information and Ferrando’s quadratic regression equations. For the purpose of this experimental research, the open-section SPP flow loop mechanism at Hydrotech (Institute of Applied Hydrodynamics and Marine Technologies, Iran University of Science and Technology) was used for model testing the propeller^26^. For the model test, the IUST model testing mechanism has been based on static multi-component balance to measure the propeller's forces and torque, and control the propeller position in three directions designed and calibrated. Using this mechanism, first, the effect of the Froude number on the hydrodynamic coefficients of the model propeller was investigated and studied its independence area within the ranges proposed by other researchers. Then the effect of positional parameters, such as immersion ratio in the range of 0.3 to 0.75, yaw angle and inclination angle of the shaft up to 10°, on the hydrodynamic coefficients and lateral forces exerted on the propeller at ten different advance ratios was investigated. Moreover, by photographing the propeller in the immersion ratio of 0.4, the development of ventilation and wake in Froude numbers 2 and 4 were analyzed. The performance test results were finally compared with the estimated values of the design phase.

## Model testing criteria for surface-piercing propellers

The ITTC Standard stipulates that the performance of surface-piercing propellers can be predicted by testing scaled models, similar to conventional propellers in water tunnels or towing tanks^27^. The model tests are designed under similarity laws between the full scale and model conditions, and the results are then generalized to the actual propeller.

However, determining test conditions and studying scaling impacts in model tests for SPP are different from conventional propellers due to the operation at the interface between water and air and the resulting increased ventilation in such propellers. To generalize the results, it is thus required to satisfy geometrical, dynamic and kinematic similarity conditions.

The basic parameters pertaining to a propeller must be considered under uniform flow and open water conditions against the advance ratio. Such coefficients can be defined in terms of the forces affecting the propeller, as follows^28,29^:

1 $$\mathrm{Advance. ratio }-J=\frac{{V}_{a}}{nD}$$

2 $$\mathrm{Thrust. coefficient }- {K}_{T} =\frac{T}{\rho {n}^{2}{D}^{4}}$$

3 $$\mathrm{Torque. coefficient }-{K}_{Q}=\frac{Q}{\rho {n}^{2}{D}^{5}}$$

4 $$\mathrm{Efficiency }-\eta =\frac{{K}_{T}}{{K}_{Q}}\bullet \frac{J}{2\pi }$$

**Figure 1:**
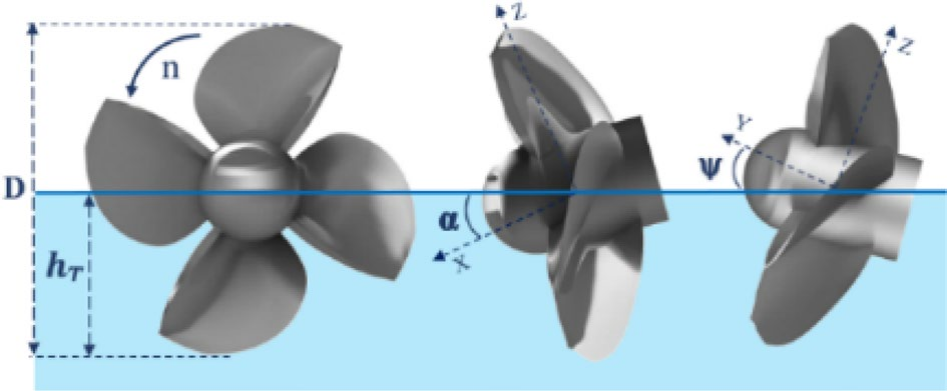
Position parameters of a SPP against free surface (Solidworks 2014, https://www.solidworks.com).

The hydrodynamic coefficients of a SPP depend on numerous parameters that can be divided into three general categories: the first includes position parameters (see Fig. 1) that relate to the position of the propeller from the free surface and direction of water flow, such as the inclination angle $(\mathrm{\alpha })$, shaft yaw $(\uppsi )$ and tip immersion ratio (${I}_{T}$).

5 $$\mathrm{Tip. Immersion. ratio }-{ I}_{T}=\frac{{h}_{T}}{D}$$

The second category is composed of geometrical parameters related to hub and blade geometries, such as diameter ($\mathrm{D}$), pitch ratio $(P/D)$, blade number ($\mathrm{Z}$), expanded area ratio ($\mathrm{EAR}$), distribution of rake angle, skew angle, and blade section. The third category includes operational parameters depending on the physics and characteristics of water flow such as velocity, ambient pressure, and the propeller's rotational speed, which are expressed as dimensionless Weber, Froude, Reynolds and cavitation numbers.

The dimensionless Weber, Froude, Reynolds and cavitation numbers are recognized as dynamic similarity parameters of propellers. Yet, it is impossible to maintain the equal similarity of all these numbers between the test model and the full-scale propeller (except for scale ratio $=1$). Researchers have offered different definitions for these numbers in terms of rotary or advance velocities of the propeller or different length parameters, and by considering the impact of each on the performance of SPPs, defined independent areas of that numbers under certain conditions, and test characteristics can thus be extracted. Unfortunately, no established global approach has been proposed yet for the precise study of SPP performance and the major relevant dimensionless parameters. The need is still felt for considering such conditions and generalizing them to different geometries^27^.

The Reynolds number's independence range has been defined in two modes by Shiba^4^ and the KSRI Institute, Russia^11^, as presented in Table 1. According to Shiba, Weber number affects the transient area and the critical advance ratio. If ${W}_{n}\ge 180$, that critical advance ratio (${J}_{\mathrm{cr}}$) will be independent of the Weber number. Brandt^8^ introduced the Weber number based on velocity (${W}_{nD}$) and considered its independence range to be 200 for all phases. Ferrando suggested the corrected Weber number equation (${{W}_{n}}^{^{\prime}}$) according to Table 1, considering the value of Weber number as dependent on geometrical characteristics, such as the blade pitch ratio^10^.

**Table 1:** Equations & ranges of dimensionless numbers effective on SPP performance.

Dimensional number	Equation and range			
Reynolds	${\mathit{Re}}_{v}=\frac{V.{c}_{0.7}}{\nu }\sqrt{1+(\frac{0.7\pi }{J}{)}^{2}}\ge 5\times 1{0}^{5}$	${\mathit{Re}}_{n}=\frac{5n{D}^{2}({A}_{E}/{A}_{O})}{\nu Z}\ge 5\times 1{0}^{5}$		
Weber	${W}_{nD}=\frac{V}{\sqrt{ \sigma / \rho D} }\ge 200$	${W}_{n}=\sqrt{\frac{\rho {n}^{2}{D}^{3}}{\sigma }}\ge 180$	${{W}_{n}}{^{\prime}}=\sqrt{\frac{\rho {n}^{2}{D}^{3}I}{\sigma }}\ge 270$	
Froude	$F{r}_{n}=n\sqrt{\frac{D}{g}} \ge 3-3.5$	${Fr}_{n{h}_{s}}=\frac{V}{\sqrt{g{h}_{s}}}\ge 4$	${Fr}_{nD}=\frac{V}{\sqrt{gD}}\ge 4$	

The Froude number is another effective parameter for propeller performance, and several studies have considered its impact on SPP performance. As mentioned in Table 1, Shiba and Olofsson introduced this number through $F{r}_{n}$ and ${Fr}_{nD}$ Equations, respectively, Based on the fixed length of the propeller diameter and the propeller's advance or rotation speed, and each defined a different independent range^4,16^. In fact, Shiba considered $F{r}_{n}<3$ to be effective within the full ventilation area, while Olofsson described the impact of ${Fr}_{nD}$ within its independence range at full and partial ventilation areas as negligible. As well, Brandt introduced Froude number ${Fr}_{n{h}_{s}}$ in terms of variable length of immersion depth, and maintained that as ineffectual in full ventilation area^8^. Ding proceeded to define $F{r}_{nD}\ge 3.5$ as the independent range of propeller behavior for all areas^12^ and the KSRI Institute similarly proposed $F{r}_{n}>3.5$ as the independence range^11^.

The cavitation number for surface-piercing propellers is defined through Eq. (6), is also known as an effective parameter on the performance:

6 $$\mathrm{Cavitation. number }-{ \sigma }_{0}=\frac{{p}_{0}-{p}_{\vartheta }}{\frac{1}{2}\rho {V}^{2}}$$

In order to control the cavitation number in model tests and obtain similarity with the full-scale conditions, it is necessary to adjust and control the pressure of water free surface with pressure control equipment. On the other hand, a number of studies have pointed to a correlation between the impacts of the cavitation number and the Froude number. Shiba introduced $F{r}_{n}\ge 3$ as the independence range for the cavitation number. Brandt, Olofsson and Ding also showed the propeller behavior within the suggested independence range to be independent of the cavitation number at numbers above 1^12,16^. The present research considered maximum independence ranges mentioned in Table 1 and determine a suitable scale for the model propeller commensurate with the relevant laboratory facilities.

These equations include the blade number ($\mathrm{Z}$), advance velocity ($\mathrm{V}$), kinematic viscosity ($\upnu$), chord length of $0.7\mathrm{R }(\mathrm{C}0.7)$, surface area ratio ($\frac{{A}_{e}}{{A}_{o}}$), surface tension coefficient ($\upsigma$), immersion depth (${h}_{s}$), and propeller rotation ($\mathrm{n}$).

## Water tunnel and propeller model testing setup

**Figure 2:**
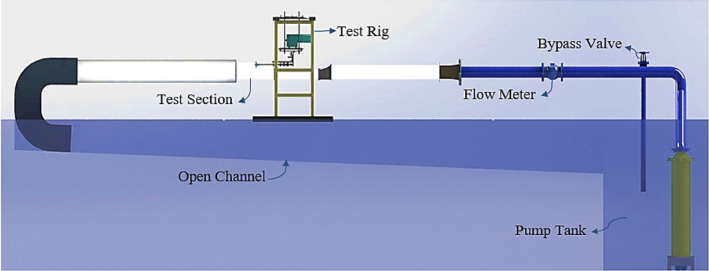
Open-section water tunnel at IUST.

Measuring the hydrodynamic forces affecting floating or submerged objects in water and studying the physics of the flow surrounding them can be counted among the most significant water tunnel applications^30^. The open-section water tunnel in the IUST was designed based on hydrodynamic studies of the flow in different areas in order to obtain suitable conditions at the test section. The tunnel (Fig. 2) was composed of an open section (250 × 200 mm), with a water velocity range of $2\mathrm{ to }10$m/s; the test section was an open area, 1.5 m long at atmospheric pressure, which hosts the propeller for test purposes, while the Plexiglass walls of this section provided for photography of the propeller water. The transfer pipeline included a bypass valve to regulate water flow velocity and a magnetic flowmeter to measure and record the instantaneous flow rate.

**Figure 3:**
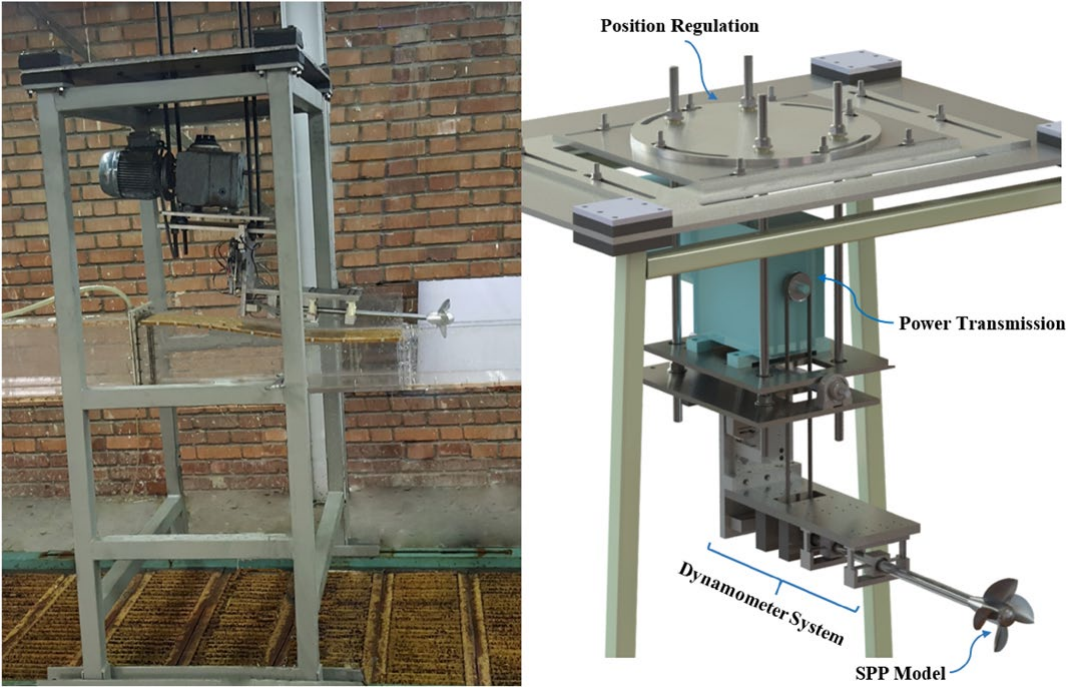
SPP test setup.

The test setup presented in Fig. 3 was designed to SPP’s test in the open-section water tunnel^26^. It included three major modules: the first was position regulation, capable of adjusting immersion depth and shaft angle against the flow direction on two horizontal and vertical planes. The system was capable of providing an inclination angle and yaw angle of $0^\circ -10^\circ$ and changed the immersion depth up to 100%. The second module is power transmission to the propeller shaft, including a gearbox motor with adjustable rev up to *3800* RPM. The engine was connected to the propeller shaft by a belt and pulley.

Measurement of the loads exerted on the SPP was conducted by the third module, using a 4-component dynamometer system based on the coordinates system on the model, and aligned with the propeller shaft. The dynamometer comprised 2-component force balances to measure lift and side forces, one 1-component balance to measure the thrust force on the propeller, and one S-type load cell to measure the propeller's reaction torque. This system was designed based on strain gauge principles and bending beam laws^31^, while the sensors also served as bearings to hold the propeller shaft and measured the supporting reaction forces at the same time^26^.

**Figure 4:**
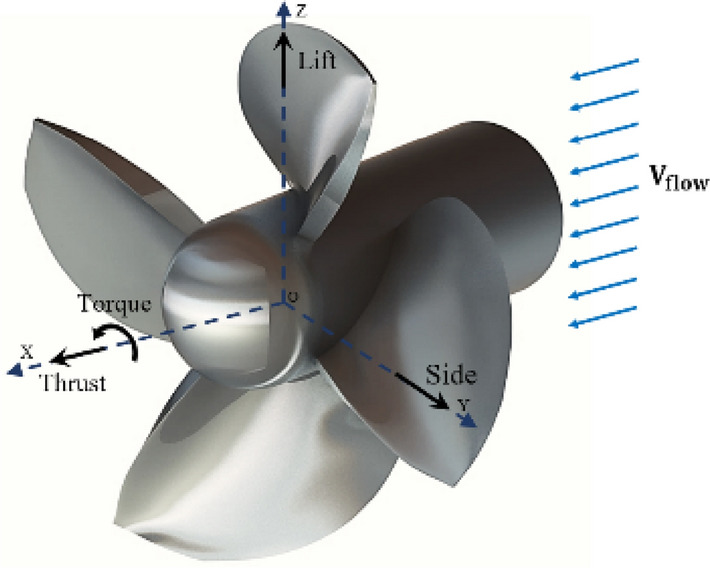
The forces and torque acting on the model propeller (Solidworks 2014, https://www.solidworks.com).

Using a six-degree-of-freedom calibration system^32^, the calibration of the test mechanism was performed within the BBD Design of Experiment. The ANOVA method was then employed to derive multivariable regression equations with a 95% confidence interval for each channel. According to Table 2, the error level of the estimated regression model for each sensor was less than 1 percent. These results show the replicability and proper performance of the system. As a result, the forces and torque acting on the model propeller (e.g. Fig. 4) in the water tunnel were directly measured through a calibration matrix. These coefficients depicted each sensor's suitable linear behavior in the direction of the design load and the existence of minimum interference among different dynamometer channels^26^.

**Table 2:** ANOVA analyses of force-torque dynamometer.

Source	Response (percent)			
Contribution	Lift	Side	Thrust	Torque
Model	99.99	99.91	99.92	99.99
Linear	99.98	99.44	99.90	99.99
Interaction	0.01	0.47	0.03	–
Error	0.01	0.09	0.08	0.01
Lack-of-fit	0.001	0.05	0.05	0.00
Pure error	0.01	0.04	0.03	0.01
R-predict	99.97	99.85	99.88	99.98

**Figure 5:**
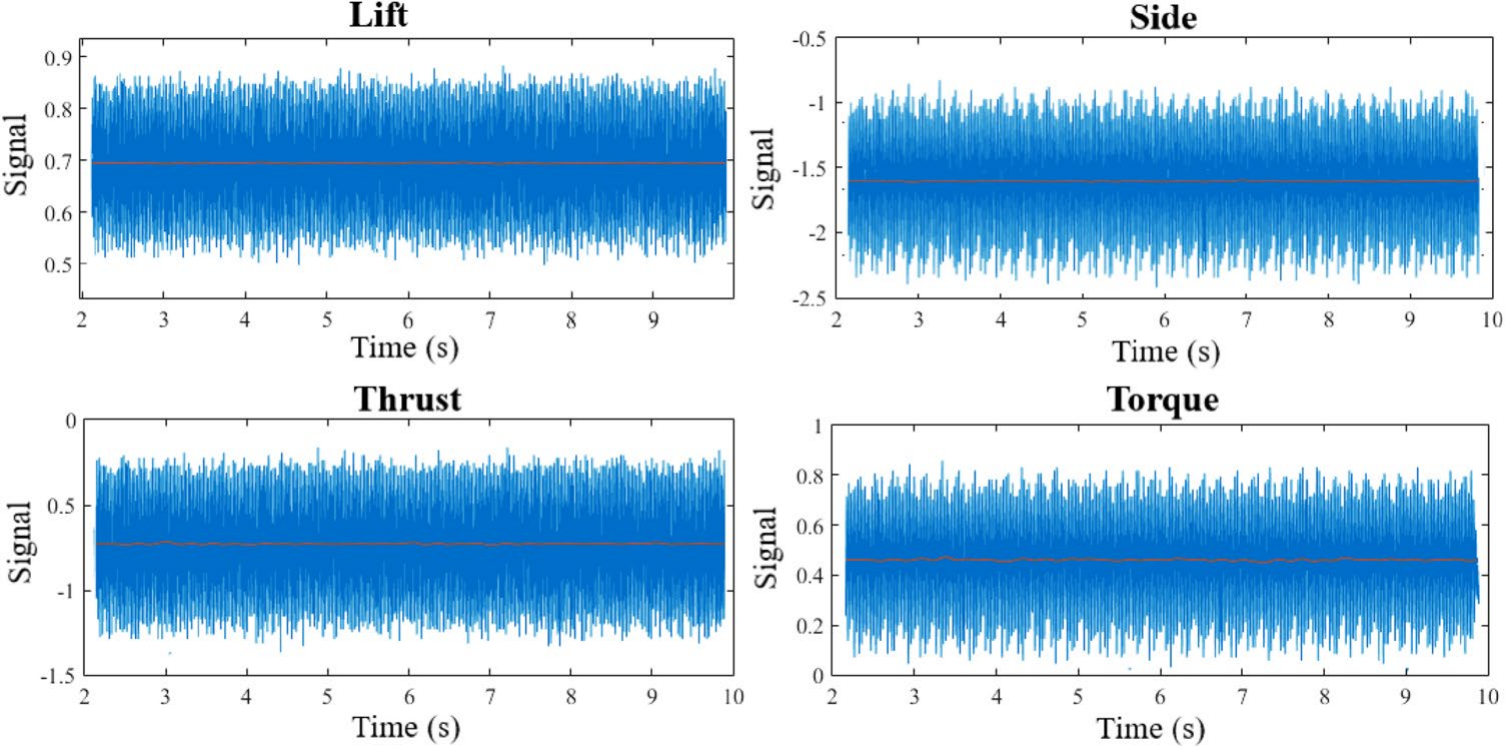
Output signals of force and torque sensors, and their filtered data.

The output signals from each sensor were recorded by a 16-channel data acquisition system, comprised of signal conditionings, amplifiers, A/D signal converters recorded (Fig. 5) with a frequency of 10 kHz at 10-s intervals. After the filtering and time-averaging process, the model propeller's average hydrodynamic load was extracted through the reverse calibration equation. The angles were measured and recorded by digital angle gauges installed on the dynamometer, with a precision of 0.1º. The immersion depth was regulated through gauges on the tunnel walls in proportion to the propeller center. Moreover, a semi-professional camera (NIKON D300) was employed for photography of the propeller during the test process in order to record and study the ventilation pattern and the resulting wake at different advance ratios. Considering the water flow advance velocity and the propeller rotational speed, photography to record the ventilation wake was conducted under a light intensity of 48,000 Lumen at ISO 1600 and shutter speed of 1/8000 s.

## Model propeller

**Figure 6:**
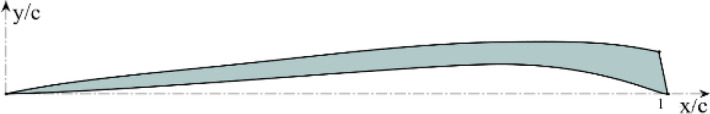
Unrolled generic blade section of HL002 SPP.

The present study used a 4-blade SPP (HL002) with a section presented in Fig. 6, designed in the IUST Hydrotech Laboratory. The design of this propeller is based on available experimental information and Ferrando’s quadratic regression equations. The geometrical specifications of the model propeller are described in Table 3.

**Table 3:** Specifications of HL002 SPP.

Propeller dimensions (HL002)			
Full scale diameter (m)	0.6	Model diameter (m)	0.13
Chord length ratio (0.7R/C)	0.422	Hub-diameter ratio	0.3
Expanded area ratio	0.58	Number of blades	4
Pitch Ratio	1.24	Propeller section type	HL002
Material (model)	AL	Turning direction	L.H

**Figure 7:**
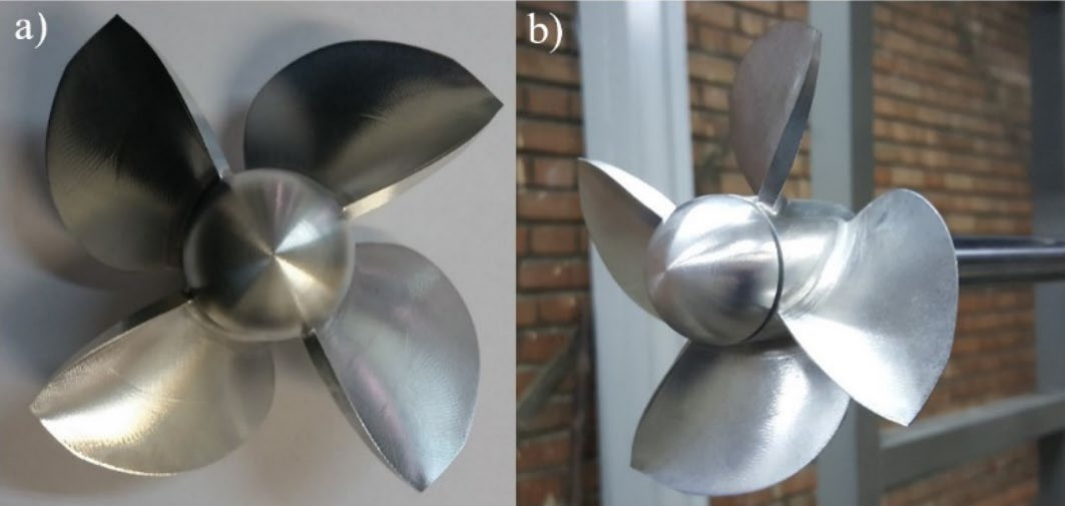
Constructed model propeller [(**a**) front view, (**b**) side view].

According to the geometrical specifications of the test section, the maximum engine rev and flow velocity in the water tunnel, the model propeller was designed at the scale ratio of $\uplambda =\frac{{D}_{s}}{{D}_{f}}=0.21$, in order to ensure similar dynamic and kinematic conditions conforming with maximum criteria in Table 1. Figure 7 portrays the constructed model propeller.

## Experimental test conditions for model propeller

For complete identification of the propeller behavior, it is desirable to extract the performance curve and study the lateral forces in a complete range of advance ratios, as designers require information to estimate the starting load and efficiency at the low advance ratios while determining the range with maximum efficiency or thrust force is necessary for high ratios. The propeller performance is also essential to be considered within the transient area due to the possible occurrence of specific vibration issues. Considering the available test setup capacities, the range $0.4\le J\le 1.4$ was selected for the present study. The tests were conducted under atmospheric pressure (non-pressurized controlled) with the priority of maximum velocity at each $\mathrm{J}$. In order to consider the impact of the Froude number, the propeller test was first conducted at immersion ratio 0.4, angle 3° from the horizontal axis and for four ${Fr}_{nD}$ in two ranges of ${Fr}_{n}$ ($F{r}_{n}=2$, $F{r}_{n}>3$), mentioned in Table 4.

**Table 4:** Range of Froude number of experimental tests.

Froude number	Test range
$F{r}_{n}$	2, > 3
${Fr}_{nD}$	2, 4, 5, 5.5

The impact of immersion ratio and inclination angle of the propeller shaft in combination was then studied for ${Fr}_{nD}=4$, according to Table 5, and finally, the impact of the propeller yaw angle against the water flow was considered at three yaw angles and the constant inclination angle at 6°.

**Table 5:** Model propeller experimental test matrix.

Variable parameter			
Test parameter	$I$	$\alpha$	$\psi$
$I$	0.3, 0.4, 0.6, 0.75	6	0
$\alpha$	0.3, 0.4, 0.6, 0.75	3, 6, 9	0
$\psi$	0.4, 0.6, 0.5	6	0, 4, 7, 10

For these tests, ${Fr}_{n}$ was considered above 3.5 at all times to eliminate the impact of the cavitation number. Under such conditions, ${\mathit{Re}}_{n}\ge 5\times 1{0}^{5}$ and the three criteria of the Weber number were considered based on advance velocity and revolution.

## Results

This section first addresses the development of ventilation and the impact of the Froude number on the propeller wake flow and flow pattern changes under advance ratios of $0.4\le J\le 1.3$, as observed through flow images. Then, the impact of changing the Froude number on efficiency and load coefficients was considered at different Froude numbers, and the impact of position parameters, such as immersion ratio, inclination angle and yaw angle at a constant Froude number was finally studied. In order to assess the possibility of using Ferrando’s quadratic regression equations in the design process, the experimental data extracted at immersion ratios of 0.4 and 0.6 were compared with the data calculated by Ferrando's Eq. (2)*.*

### Ventilation and flow regime

**Figure 8:**
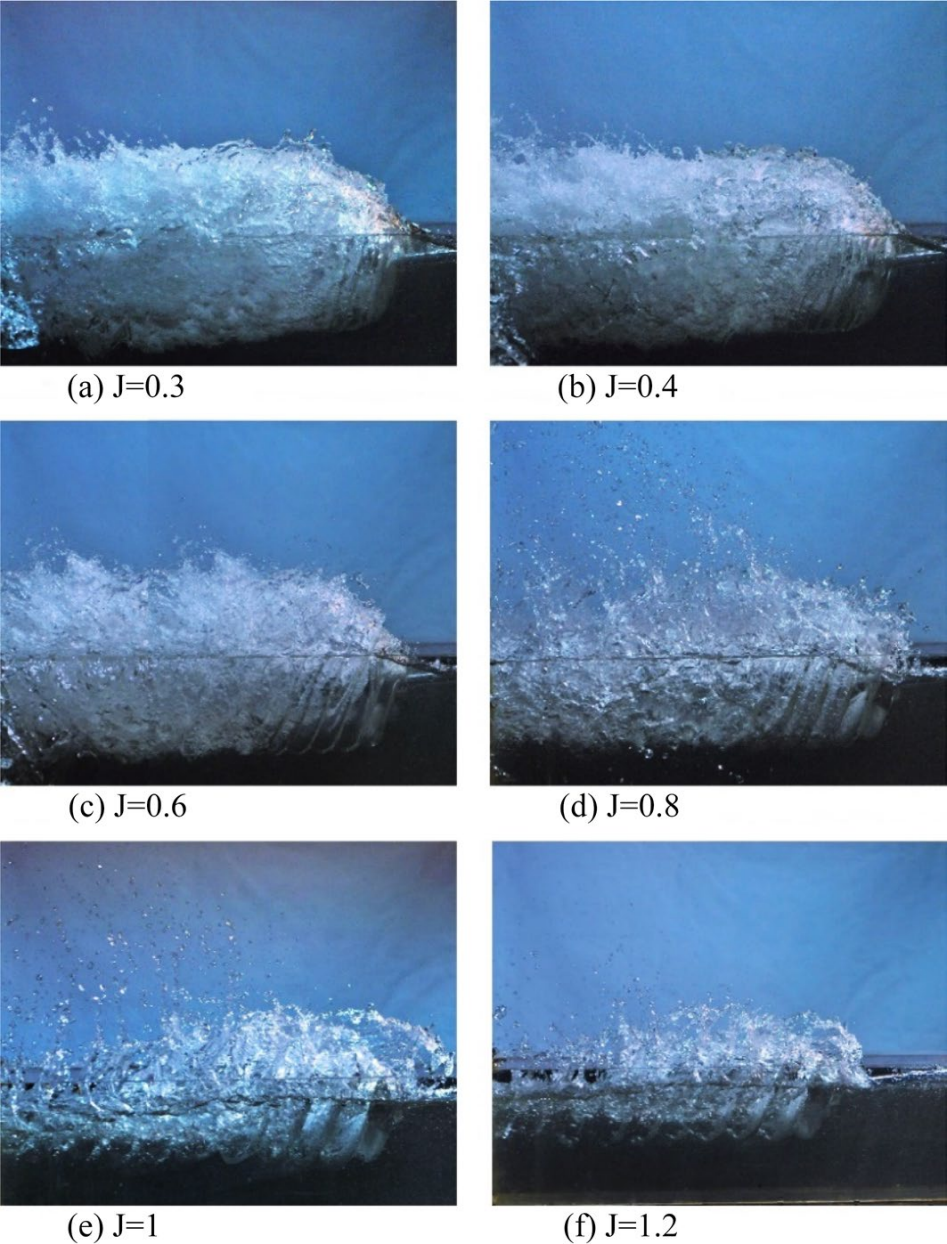
Flow pattern and ventilation development in $F{r}_{nD}=2$.

**Figure 9:**
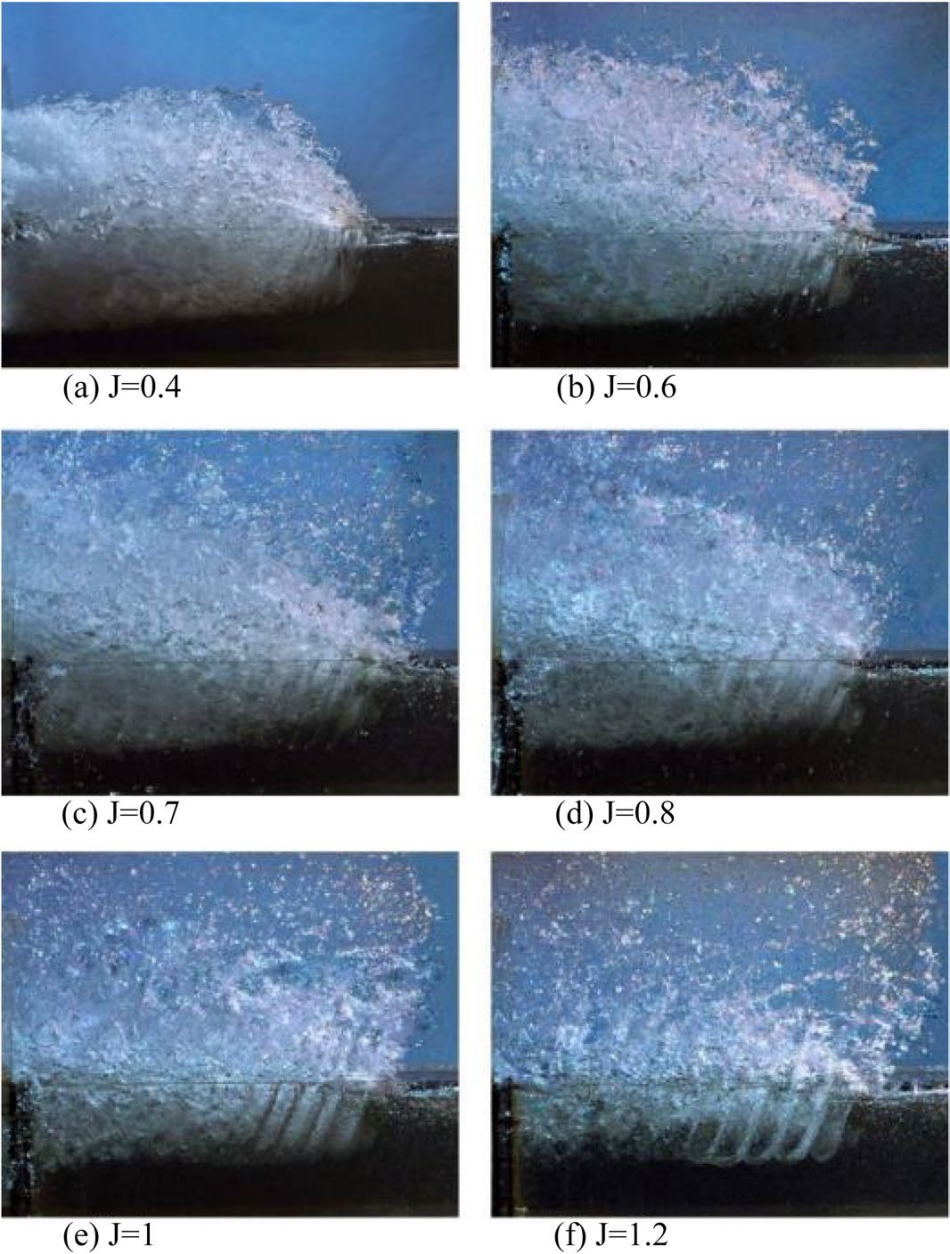
Flow pattern and ventilation development in $F{r}_{nD}=4$.

By comparing Figs. 8 and 9 that show the water flow passing the propeller at advance ratios 0.3 to 1.2 and Froude number (${Fr}_{nD}$) of 2 and 4, one could vividly observe the developed propeller ventilation with the reduction of the advance ratio.

At $\mathrm{J}=1.2$ and thrust close to zero (Fig. 8f), the amount of air following the blade below the water surface is tiny at first, and the wake only forms as a vortex at the blade tip, while the entire surface of the blade is entirely wet. Here, the spray of water is also low, and the water surface remains almost undisturbed. With the reduction of the advance ratio to $\mathrm{J}=1$, the ventilation vortex grows and stabilizes, and the wake layers form closer to one another (Fig. 8e). Under such a flow regime, the cavity vortex sheets are visibly separate, and the water volume lies between them, while the water spray in the air also increases.

A significant change in the flow pattern is observed with the further reduction to the advance ratio coefficients to $\mathrm{J}=0.8$ (Fig. 8d). At higher rotation velocities, the suction behind the blades sucks more air into the water. The cavitation vortexes grow in a manner that the wake layers collide and dissipate more rapidly in the downstream area. Here, extensive water spray can be observed, and the water level begins to rise in front of the propeller. Under such conditions of the propeller, known as the transient area,${K}_{Q}$ and ${K}_{T}$'s values are not unique for the given advance ratio.

For advanced coefficients of 0.6 and below (Fig. 8c), the ventilation cavity is developing on the blade's backside, leading to higher cavity diameter, rising water level, and increased immersion surface. Under full ventilation, the cavity's volume moving downstream grows, and a thin layer of water runs between bulky cavity layers. Due to such developments, the cavity layers collide and dissipate more quickly, while the volume of water sprayed into the air also increases. When the advance ratio reaches 0.3 or 0.4, the cavity attached to the blade backside thickens significantly (Fig. 8a,b). It blocks water from passing through the blades, thus reducing the flow through the propeller and increasing the flow around it. Such a phenomenon lowers the thrust created by the propeller. This explains the reduction of ${K}_{Q}$ and ${K}_{T}$ at lower advance ratios.

By comparing the flow pattern formed under similar advance ratios for Froude numbers 2 and 4 (Figs. 8 and 9), the impact of the Froude number on the ventilation cavity and wake can be observed, as the cavity is shorter and the wake diameter reduces downstream for the smaller Froude number. At all advance ratios, the cavity layer diameter, as well as volume and range of water spray, grow with increased Froude numbers, while the wake pitch and pitch angle reduced. It could thus be concluded that the impact of the Froude number is visible for all ventilation areas. Yet, it left a more significant impact on the transient and partial ventilation areas.

### Froude number

**Figure 10:**
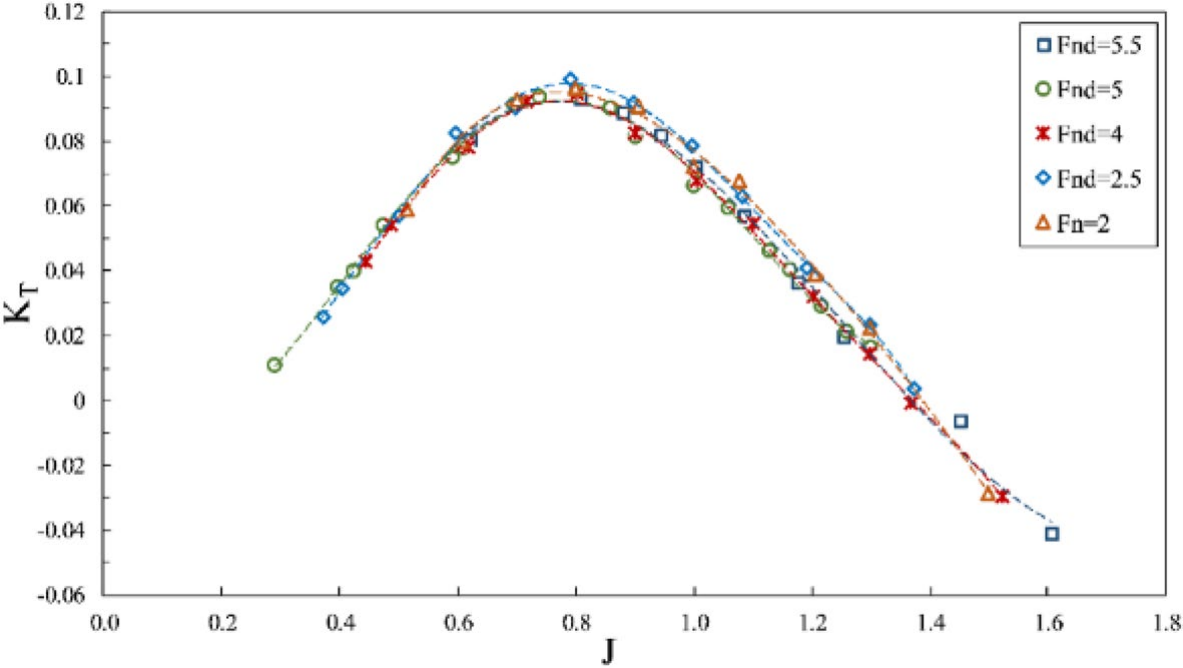
Thrust coefficient in propeller direction in different Froude numbers ($I=0.4,\alpha =3^\circ$).

**Figure 11:**
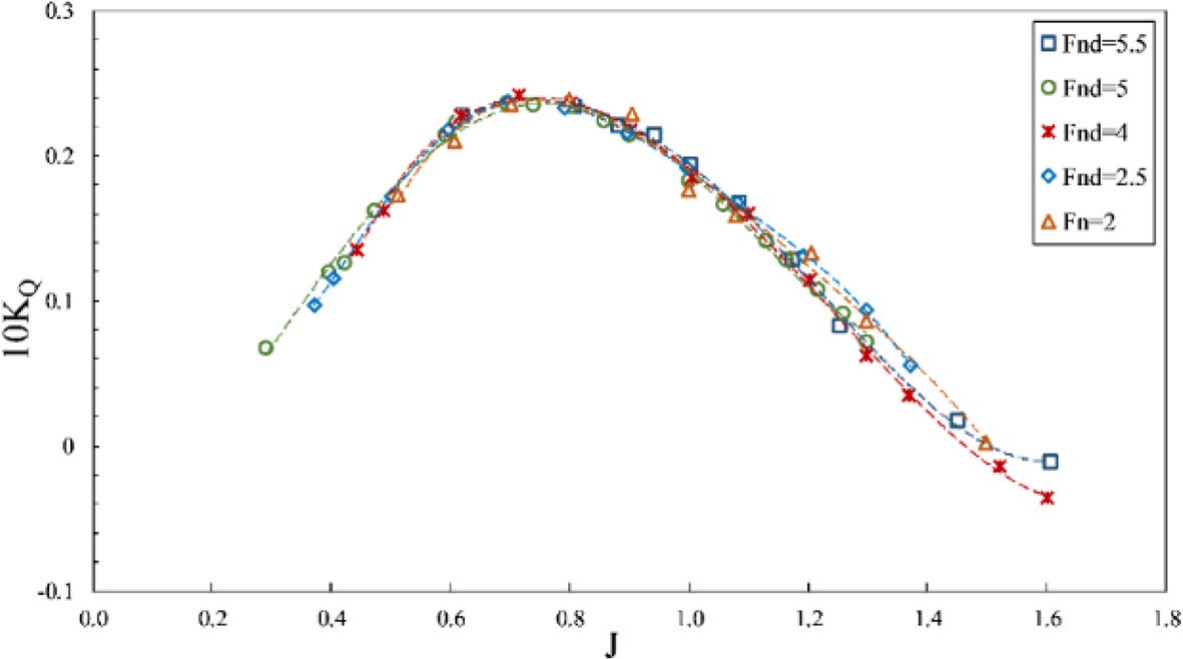
Torque coefficient in propeller direction in different Froude numbers ($I=0.4,\alpha =3^\circ$).

**Figure 12:**
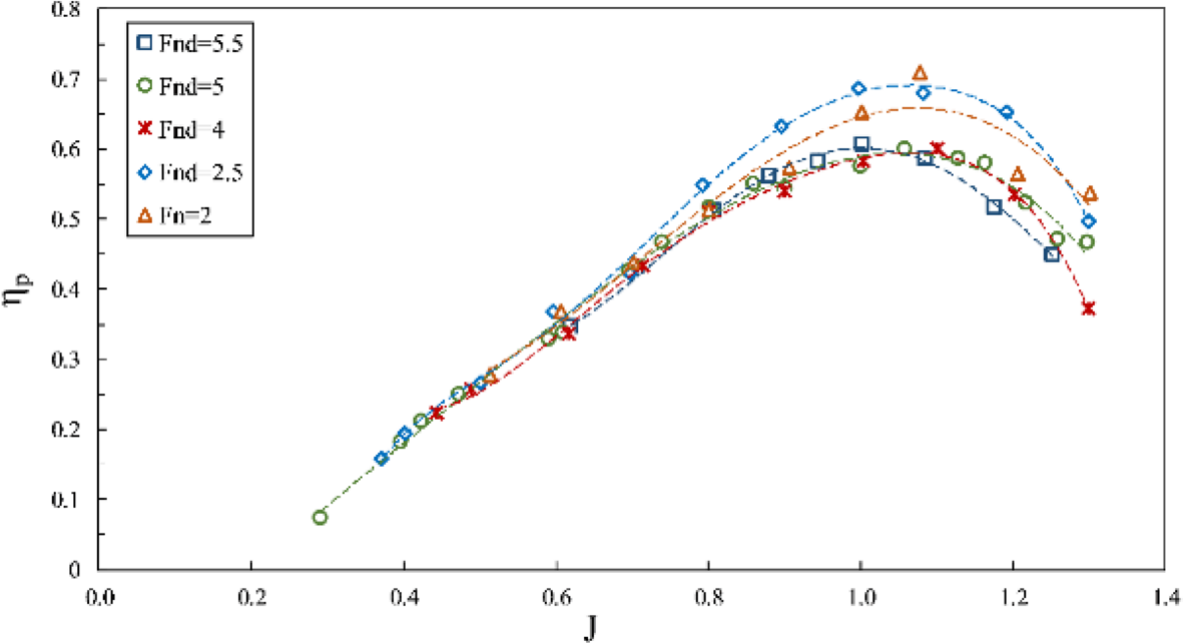
Efficiency in propeller direction in different Froude numbers ($I=0.4,\alpha =3^\circ$).

The impact of the Froude number on propeller performance can be observed in the data from Figs. 10, 11 and 12, which depict thrust coefficient, torque coefficient and propeller efficiency, respectively, at a constant immersion ratio of 0.4 in propeller shaft direction. In these figures, the force and torque coefficients of the propeller are compared in three conditions: (1) data with $F{r}_{n}=2$ and constant rotational speed, where flow velocity changed the ${Fr}_{nD}$ between 1.7 and 2; (2) test data with $F{r}_{nD}=2$ and constant advance velocity and variable rotation; and (3) data with ${Fr}_{nD}\ge 4$, under the requirement of $F{r}_{n}\ge 3$. Comparing the propeller behavior under these conditions shows higher thrust coefficients for tests with ${Fr}_{nD}\le 2$, visible at all areas but less effective under full ventilation. For tests with ${Fr}_{nD}\ge 4$, the full ventilation and transient areas displayed propeller performance and efficiency as slightly dependent on the Froude number. In contrast, the Froude number's reduction under partial ventilation results in higher ${K}_{T}$ and increases up to 30 percent under certain advance ratios. Higher Froude numbers do not lower the thrust coefficient under full ventilation, while slight increases have also been observed due to high ventilation volume around the propeller in this area. At higher Froude numbers, the cavity moves with higher speed, and that improves the performance.

**Figure 13:**
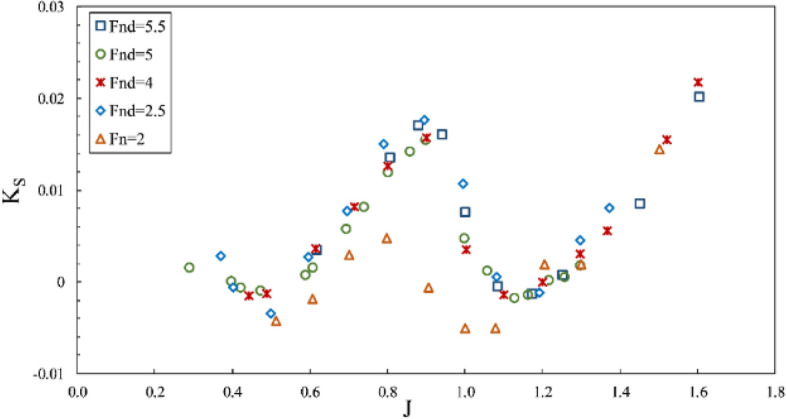
Side force coefficient in propeller direction in different Froude numbers ($I=0.4,\alpha =3^\circ$).

**Figure 14:**
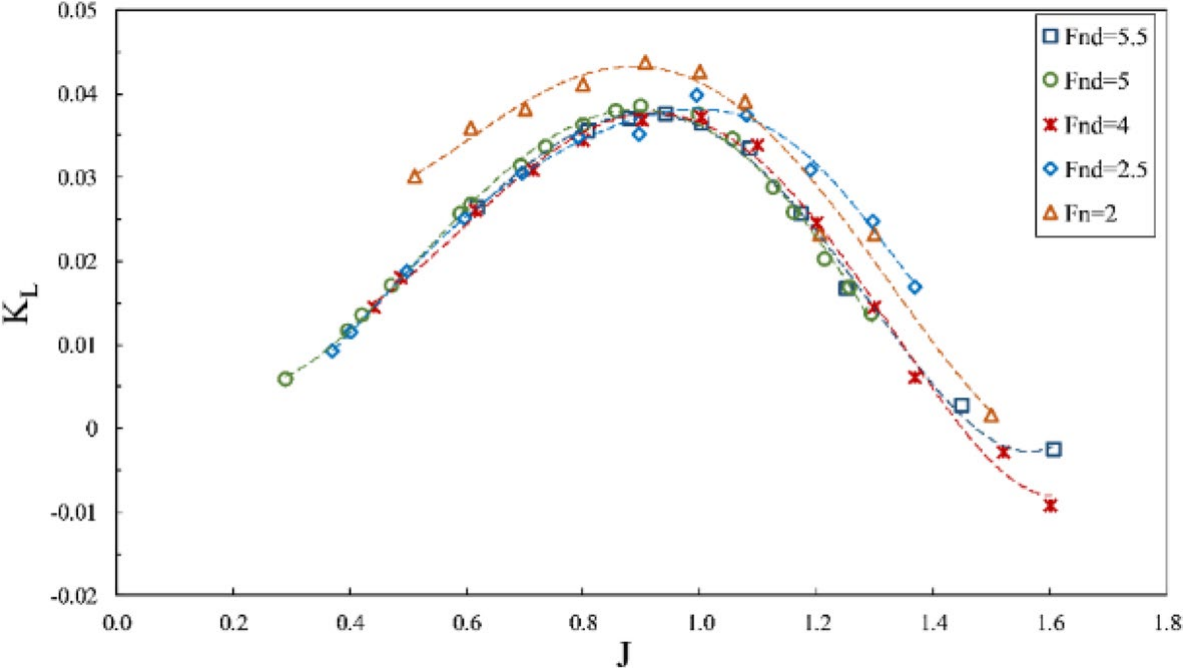
Lift force coefficient in propeller direction in different Froude numbers ($I=0.4,\alpha =3^\circ$).

However, the torque coefficient is little affected by changes in the Froude number, and tangible changes are observed only under partial ventilation. Comparing the propeller performance curve at $F{r}_{n}=2$ with tests for $F{r}_{n}\ge 3$ shows that the thrust coefficient behaves differently in all areas, especially under full ventilation, which reveals the impact of the independence range for this number ($F{r}_{n}\ge 3$) on hydrodynamic coefficients and efficiency of the propeller. In Figs. 13 and 14, it is evident that sensitivity reduces at ${F}_{nD}\ge 4$, and the changes are not significant. Nevertheless, side and lift forces vary extensively at other Froude numbers, which points to different flow patterns.

Finally, it can be concluded through force coefficient data and propeller efficiency that ${F}_{nD}\ge 4$ and $F{r}_{n}\ge 3$ can be identified as the independence range under transient and full ventilation, while the Froude number affects the propeller performance and efficiency within the partial ventilation area.

### Immersion ratio

This ratio is one of the critical parameters effective in the design and performance of surface propellers. The shaft inclination angle, the vessel's trim and the propeller behavior under different advance ratios affect the immersion level, and this parameter cannot thus be precisely controlled. Immersion tests were conducted at different shaft inclination angles to study the impact of this parameter separately.

**Figure 15:**
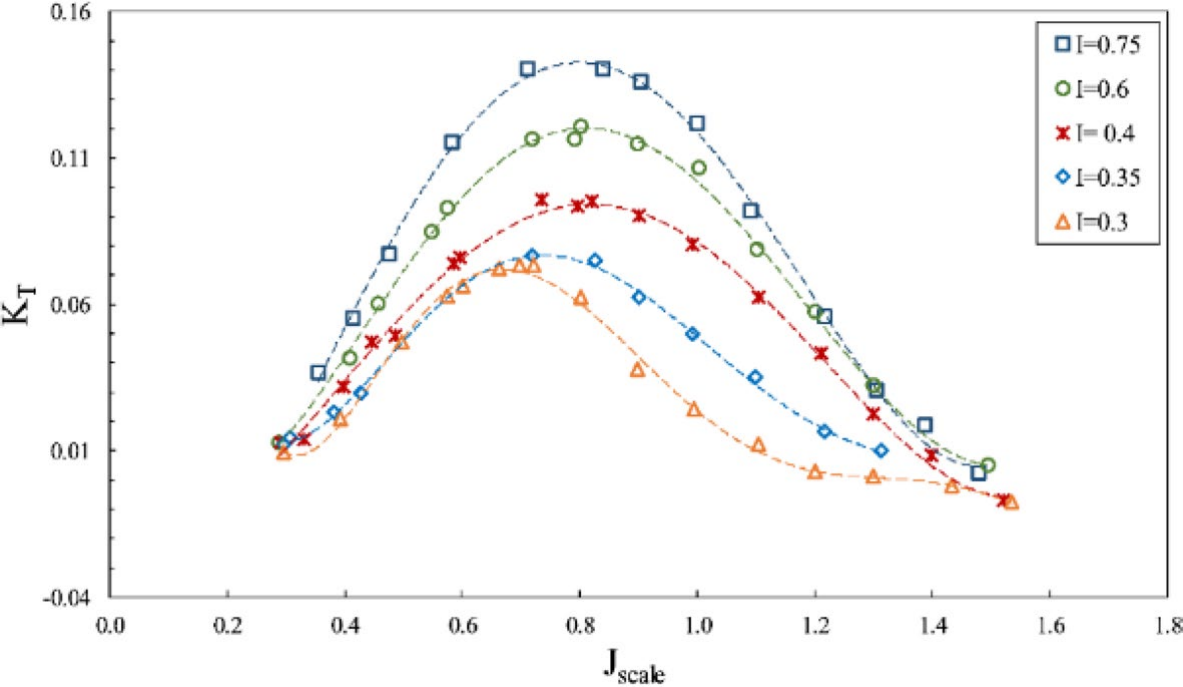
Effect of immersion ratio on the thrust coefficient in the propeller direction ($\alpha =6^\circ$).

**Figure 16:**
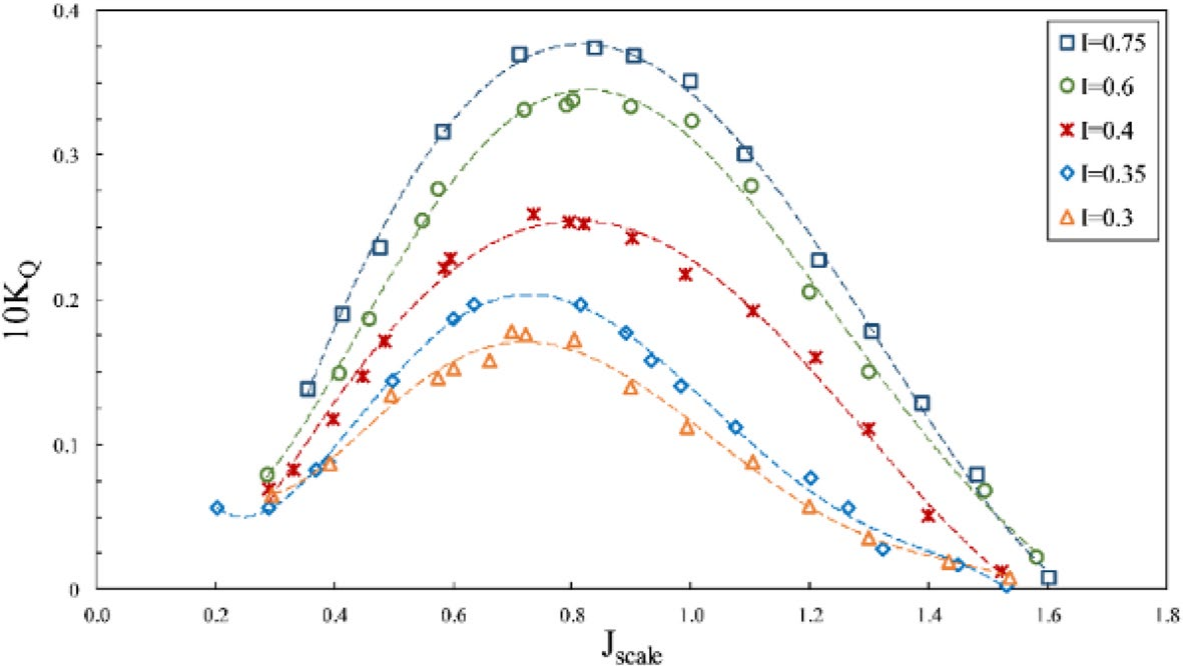
Effect of immersion ratio on the torque coefficient in the propeller direction ($\alpha =6^\circ$).

**Figure 17:**
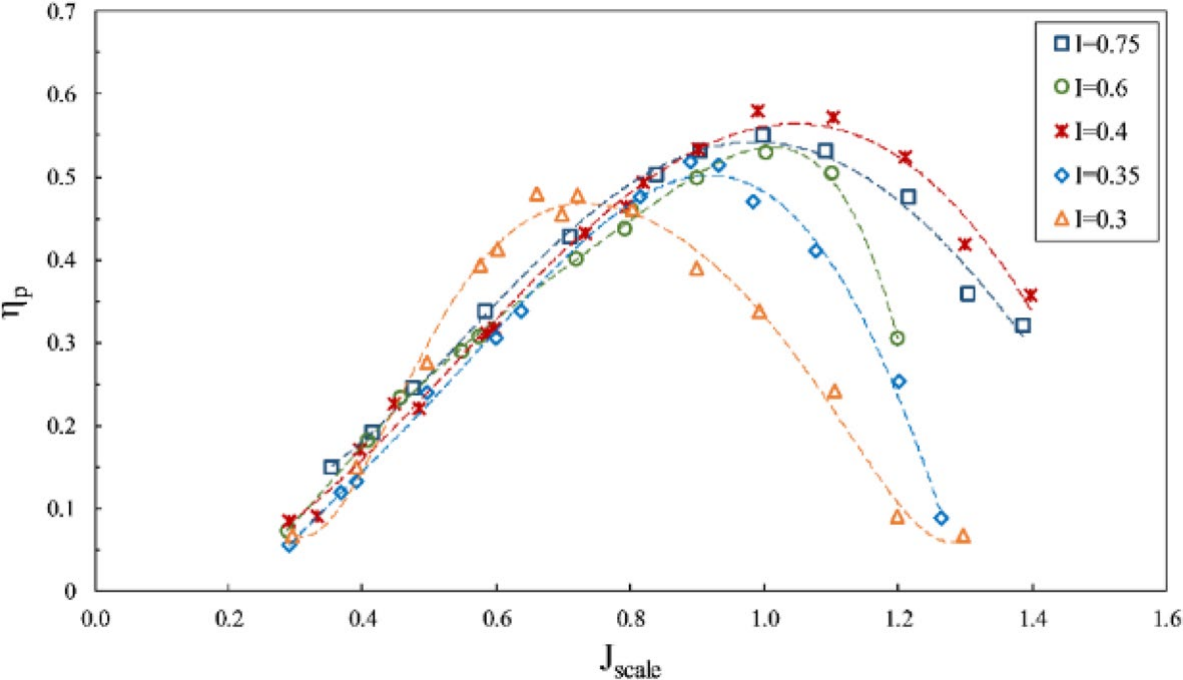
Effect of immersion ratio on the efficiency in the propeller direction ($\alpha =6^\circ$).

According to Figs. 15, 16 and 17, where the impact of immersion change was considered at the constant angle of 6º and for constant coordinates of the propeller shaft, the advance ratios (${J}_{scale}$) in these figures was obtained in terms of advance velocity in line with the shaft. Reduced immersion ratio will in fact affect propeller effective disc area and developed ventilation cavity behind the propeller. These two parameters influence the lift and drag exerted on the propeller and affect the thrust and torque at any advance ratios. The efficiency will thus change commensurate with the thrust to torque changes. In addition, reducing the wet area of the SPP also reduces drag and can improve propeller efficiency.

As evident in Figs. 15, 16 and 17, reduced immersion depth is followed by generally lower thrust and torque coefficients due to decreased propeller submerged area. However, their changes as affected by the ventilation and wet area of the propeller is not similar. At advance ratios above the critical advance ratio ($J>0.8$), increased immersion depth from 0.3 to 0.75 left a high impact on changes of the torque coefficient, yet the thrust coefficient does not display similar behavior in different advance coefficients. Such changes point to different ventilation development behind the blade at different immersion depths. Within this range of advance ratio, the maximal efficiency occurs at an immersion depth of 0.4, due to the reduced torque of the propeller at this depth compared to higher depths and the limited thrust changes.

With developed total ventilation at low advance ratios ($J<0.8$), thrust changes of the propeller will be limited at immersion depths of 0.3 to 0.4, and lower than torque reduction, meaning that the propeller efficiency will be higher at immersion depth of 0.3 than higher depths. This is while with the increased advance ratio at this depth (${\mathrm{I}}_{\mathrm{T}}=0.3$), the efficiency will fall drastically due to extensive reduction of propeller thrust.

Finally, experimental considerations identified the maximal efficiency of the SPP with a suitable thrust to occur at $0.4<{\mathrm{I}}_{\mathrm{T}}<0.75$ and the advance ratio of 0.9 to 1.1. The maximum thrust coefficient at the immersion ratio of 0.75 and $J=0.8$ was measured at 0.14, and maximum efficiency at the immersion ratio of 0.4 and $J=1$ occurred at 58%.

**Figure 18:**
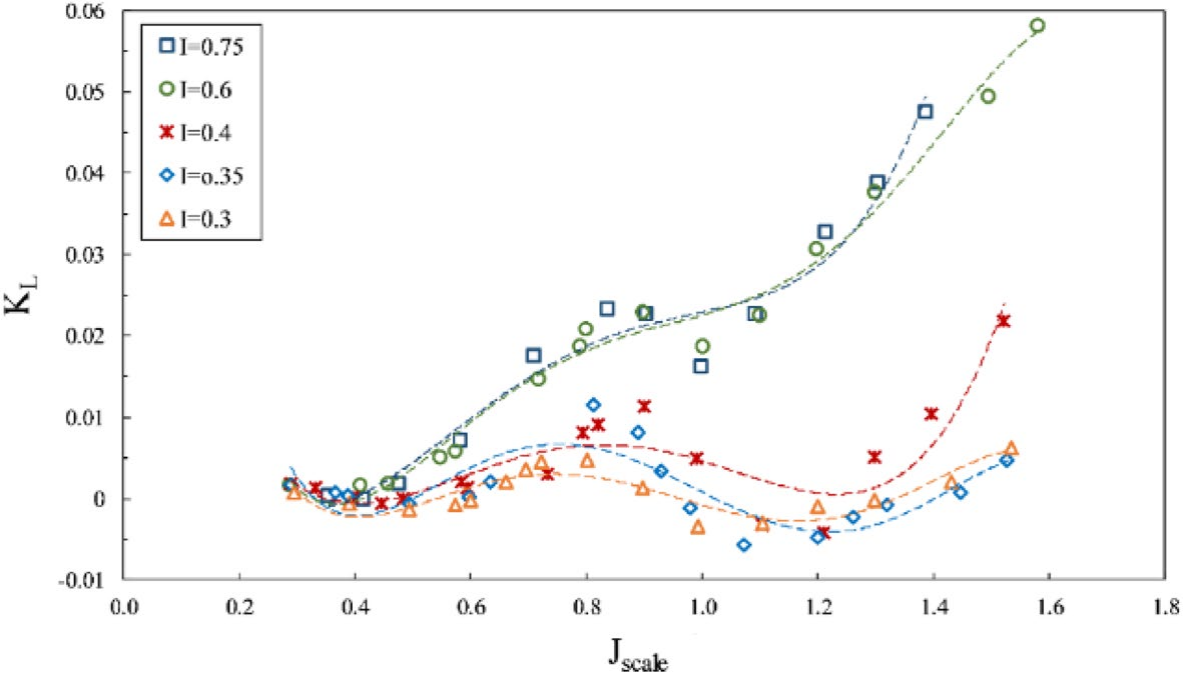
Effect of immersion ratio on the lift coefficient in the propeller direction ($\alpha =6^\circ$).

**Figure 19:**
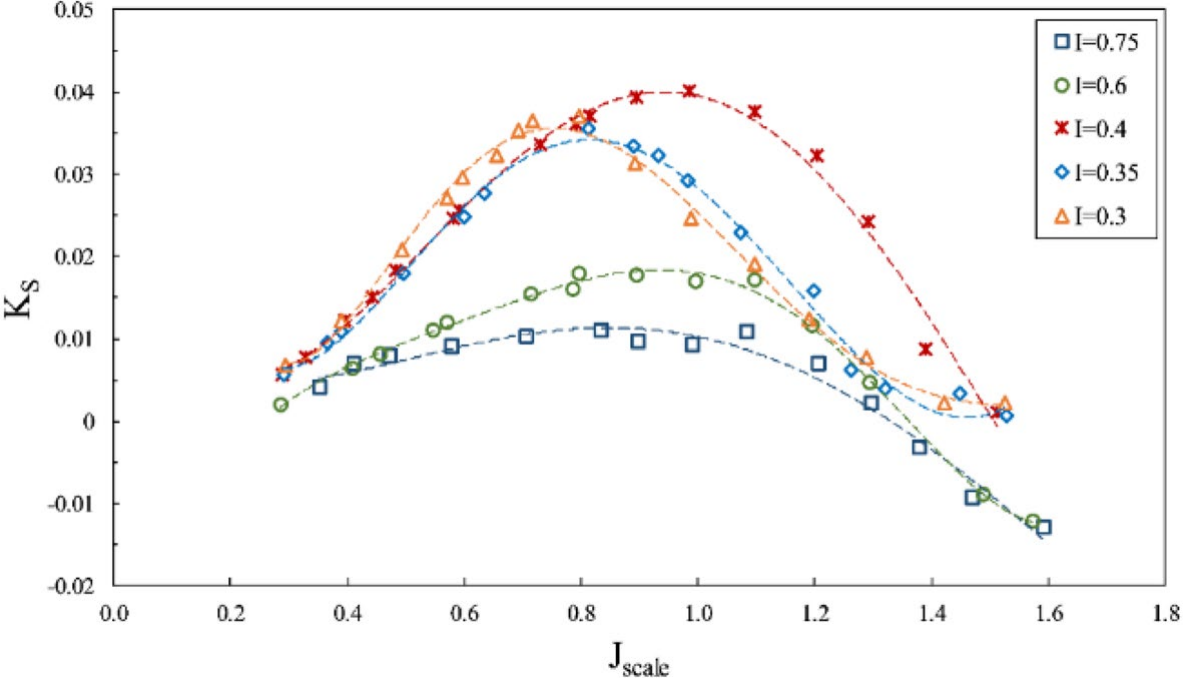
Effect of immersion ratio on the side force coefficient in the propeller direction ($\alpha =6^\circ$).

The lateral forces were considered in Figs. 18 and 19. According to the results, vertical force on the shaft was generally upward, unless the amount of forces was low when the aggregate vertical force would be downward, due to extensive water spray and agitation. Figure 18 shows the lift coefficient to rise with increased immersion at all advance ratios, while the side force coefficient (Fig. 19) reached its pinnacle at low immersion ratios and tended to rise with decreased immersion ratio. This difference occurs because, at a low immersion ratio, the blade tip serves as the effective part in generating thrust, where the aggregate forces would be more oriented toward the horizontal component rather than the vertical one.

In general, comparing the coefficients of lift and yaw forces revealed a higher generation of horizontal forces than the vertical ones and the changes accompanied by thrust. However, the lift coefficient showed different behavior in the transient area due to drastic load fluctuations in this area. The ventilation over the blade surface vastly changes in this area and thus moves the load center on the blade.

### Shaft inclination angle

Due to their asymmetrical operational conditions and fluctuations of the resulting wake in SPP, significant lateral forces are produced. It is expected that by changing the shaft angle, the resultant forces become aligned with the vessel's advance and result in increased efficiency in that direction.

**Figure 20:**
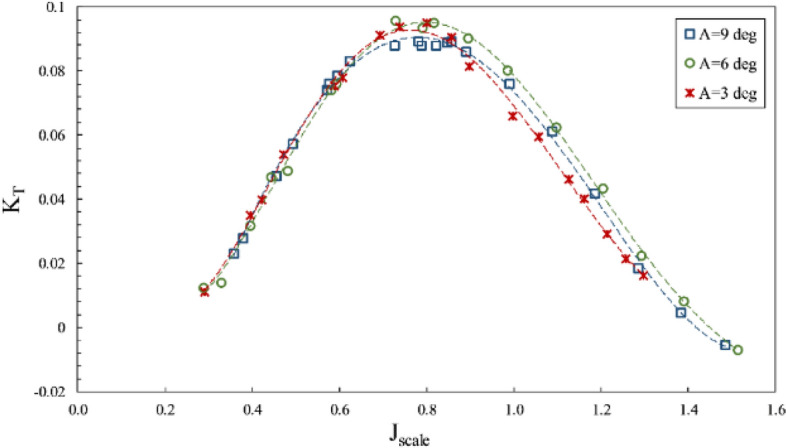
Thrust coefficient on propeller direction in different inclination angles ($I=0.4$).

**Figure 21:**
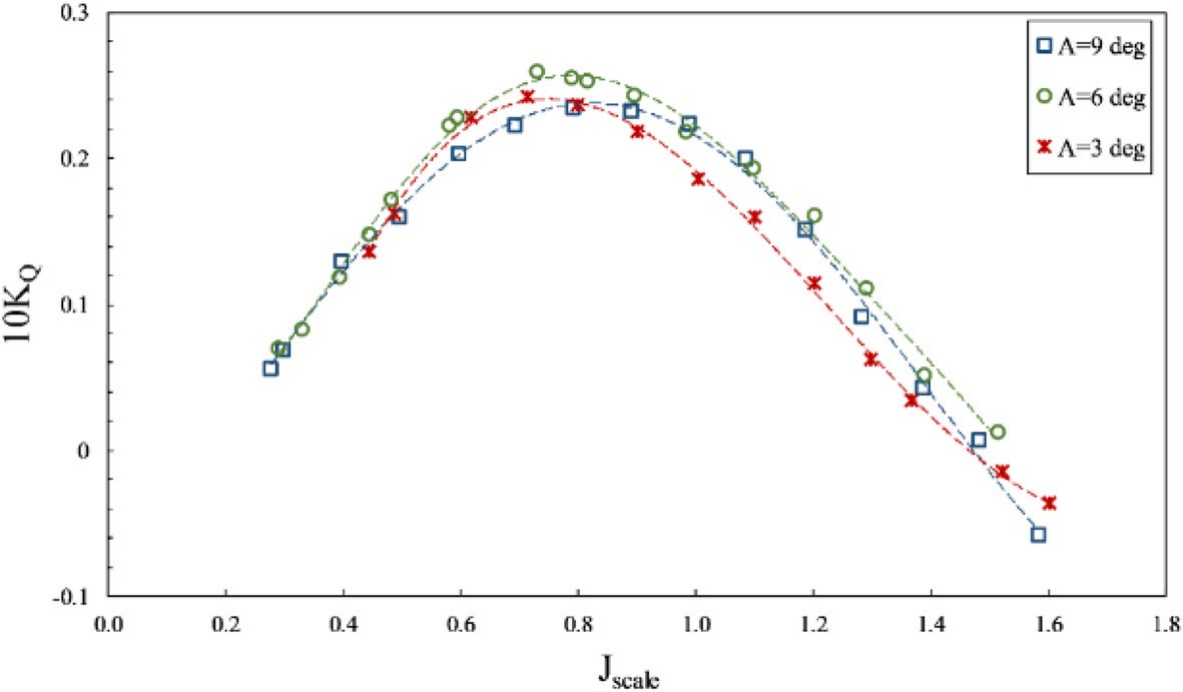
Torque coefficient on propeller direction in different inclination angles ($I=0.4$).

**Figure 22:**
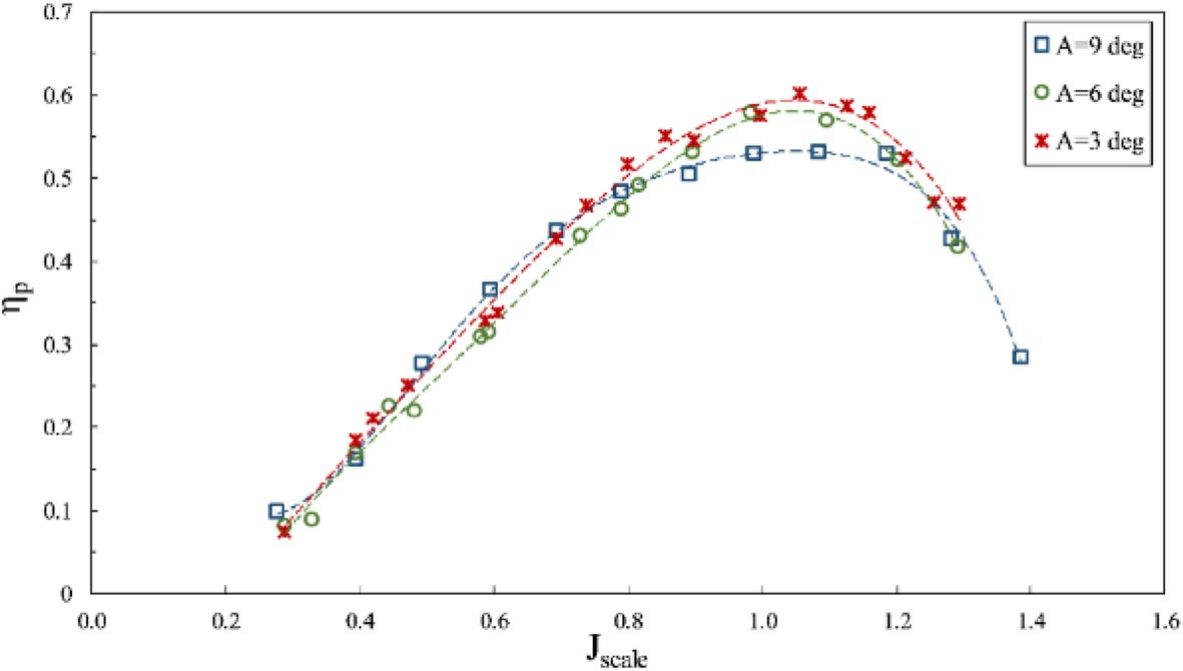
Efficiency on propeller direction in different inclination angles ($I=0.4$).

The impact of changes in the inclination angle for the SPP on thrust and torque coefficients and efficiency against fixed shaft (in line with the propeller shaft) coordinates was considered at the constant immersion ratio of 0.4 and displayed in Figs. 20, 21, and 22, respectively.

According to Figs. 20 and 21, an increase in the inclination angle changed torque and axial thrust coefficients. The impact of increased inclination angle is higher at the partial ventilation area ($\mathrm{J}>0.8$), yet its effect on the thrust reduces with cavity development at the total ventilation area ($J<0.8$). Such change due to angle increase from 3º to 6º reached its highest level of 40 percent for $1<J<1.2$, Nevertheless, increasing the angle to 9 º has reduced the thrust coefficient in most advance coefficients. Figure 22 illustrates that angle increases are not always associated with an improvement in propeller performance, and combining propeller position relative to the free surface and the ventilation cavity behind the propeller can produce different results at various angles. Consequently, in order to determine the optimal position of the SPP, the optimal angle must be determined experimentally.

**Figure 23:**
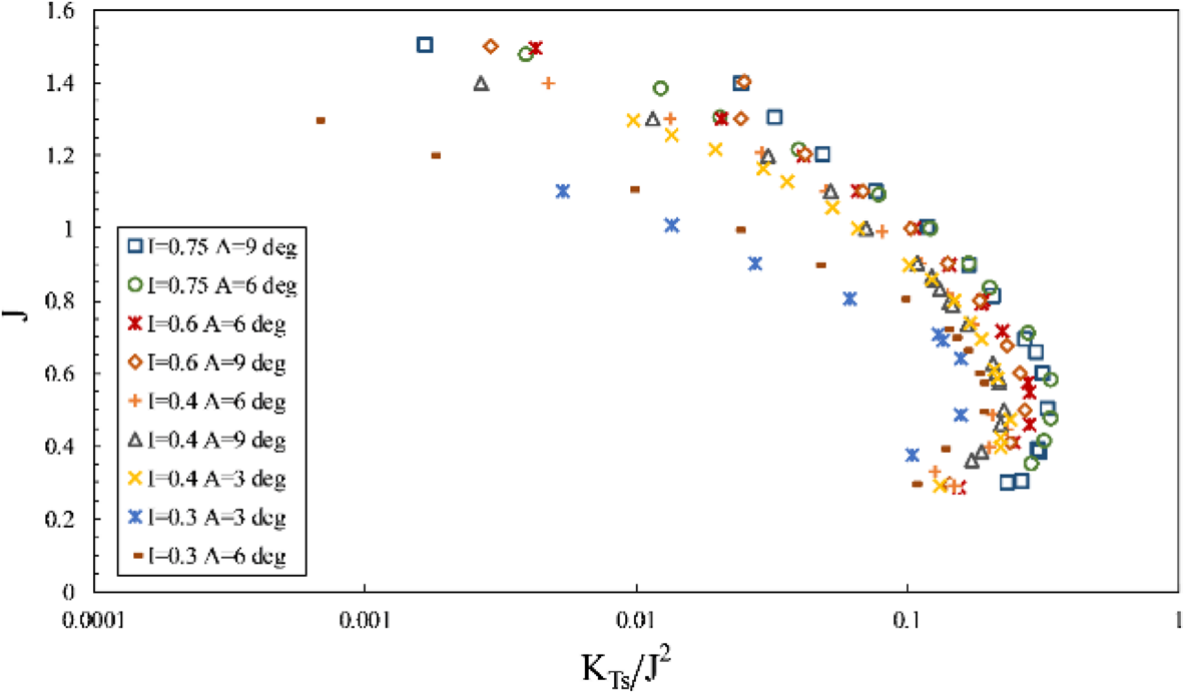
Variation of thrust coefficient in the propulsion direction at different immersion ratios and inclination angles.

**Figure 24:**
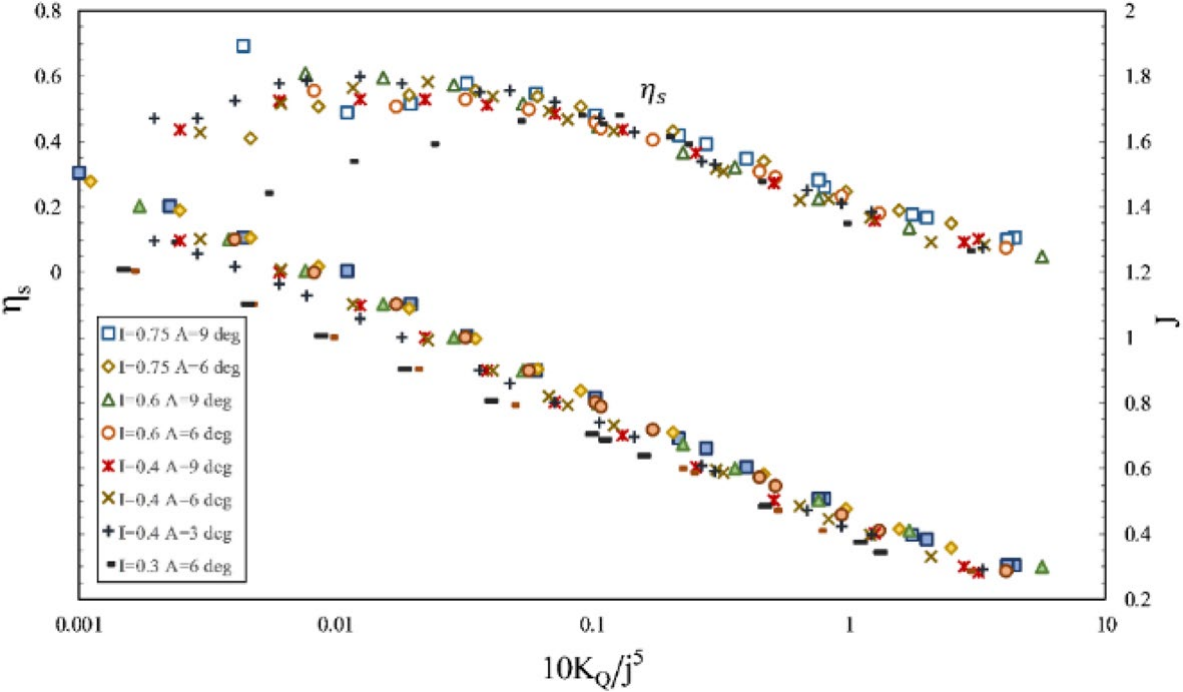
Variation of torque coefficient in the propulsion direction at different immersion ratios and inclination angles.

Furthermore, the impact of inclination angle change on performance in line with the vessel's advance was considered by comparing thrust and torque coefficients and efficiency against fixed shaft coordinates (Figs. 23 and 24) at different immersion ratios and angles. The thrust and torque coefficients in line with the vessel's advance were expressed by *K*_*TS*_/*J*^2^ and *K*_*QS*_/*J*^5^, which define actual values of force change as independent from velocity and diameter, comparable with other propellers. According to the figures data, the propeller's hydrodynamic coefficients are more effective from immersion ratios and improved with increased shaft inclination angle. The angle 6º was identified as the optimum shaft inclination angle among propeller efficiencies at each immersion ratio. The maximum level of *K*_*TS*_/*J*^2^ coefficient was realized at immersion ratio of 0.75, shaft inclination angle of 6º and advance ratio of 0.6, which indicates a favorable effect of increasing immersion depth and angle on the vessel's advance force. The data in Fig. 24 and comparison of changes in *K*_*QS*_/*J*^5^ at different advance ratios also showed that the range of propeller reaction torque changes increased with higher advance ratios, denoting a higher impact of position parameters, namely inclination angle and immersion under partial ventilation. Moreover, the efficiency data in line with the vessel based on the *K*_*QS*_/*J*^5^ coefficient depicted higher changing capacity of efficiency with position parameters at lower load coefficients.

**Figure 25:**
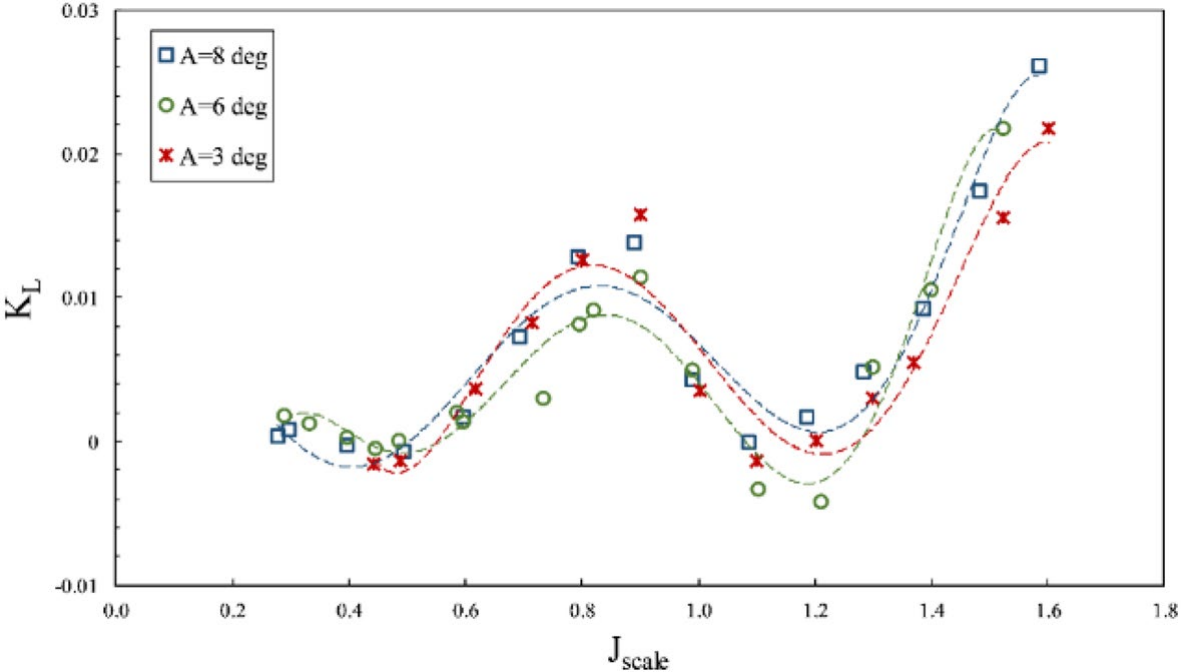
Lift coefficient on propeller direction in different inclination angles ($I=0.4$).

**Figure 26:**
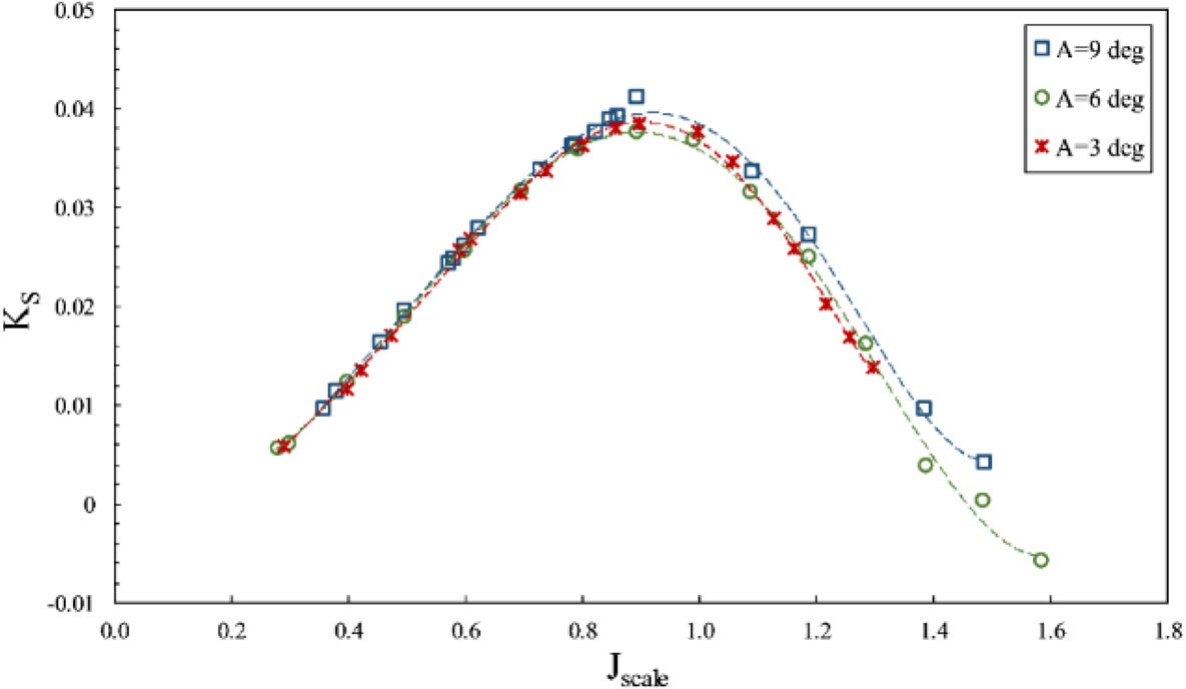
Side force coefficient on propeller direction in different inclination angles ($I=0.4$).

The effective side forces were studied at different angles, according to Figs. 25 and 26. The lift coefficient chart pointed to increased vertical forces on the shaft at constant coordinates, which needs to be considered in designing the shaft bearing and support. The side force coefficient also remained unchanged, according to Fig. 26, and the replicable form of this force confirmed the compatibility and replicability of this testing system.

### Shaft yaw angle

According to the data gathered for the side force coefficient, the effective horizontal force counted for a more significant percentage in the tests than the vertical force, reaching 40% of the length thrust at its maximum. From a theoretical point of view, the shaft deviation to the side and deviation from the vertical plane could be adopted for using the maximum resultant force along the advance direction. In fact, a yaw angle suitable for the SPP would have a lower side drag. It could serve as an appropriate solution to promote propulsion efficiency without any damage or risks resulting from vibration or strength issues. However, this potential advantage needs to be tested, and the level of efficiency loss resulting from the hydrodynamic impacts of geometries and other parameters determined.

**Figure 27:**
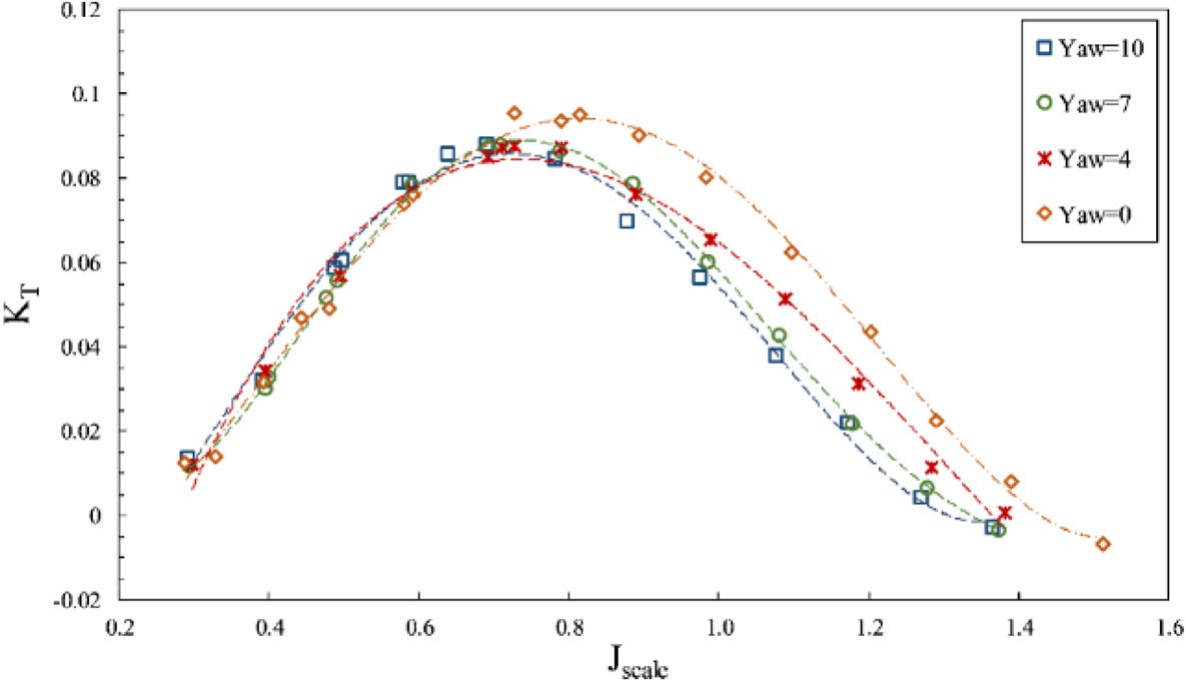
The effect of yaw angle on the thrust coefficient in the propeller direction ($I=0.4$).

The present study considered the yaw angle up to 10º at two immersion ratios of 0.4 and 0.6. By observing the thrust coefficient aligned with the shaft at the mentioned immersion ratios, such as Fig. 27, the shaft axial thrust was reported to diminish with increased yaw angles. The changing angle from the horizontal axis reduced the effective pitch of the propeller for sweeping the water. Due to the differing orientations of propeller and flow, the propeller's swept area was reduced when portrayed perpendicularly (aligned with the flow). Such reduction was more intense at lower immersion ratios because of the force center movement toward the blade tip and higher susceptibility of lateral forces from the shaft angle. These changes are higher at the partial ventilation area, and its impacts decrease with cavity development at the total ventilation area.

**Figure 28:**
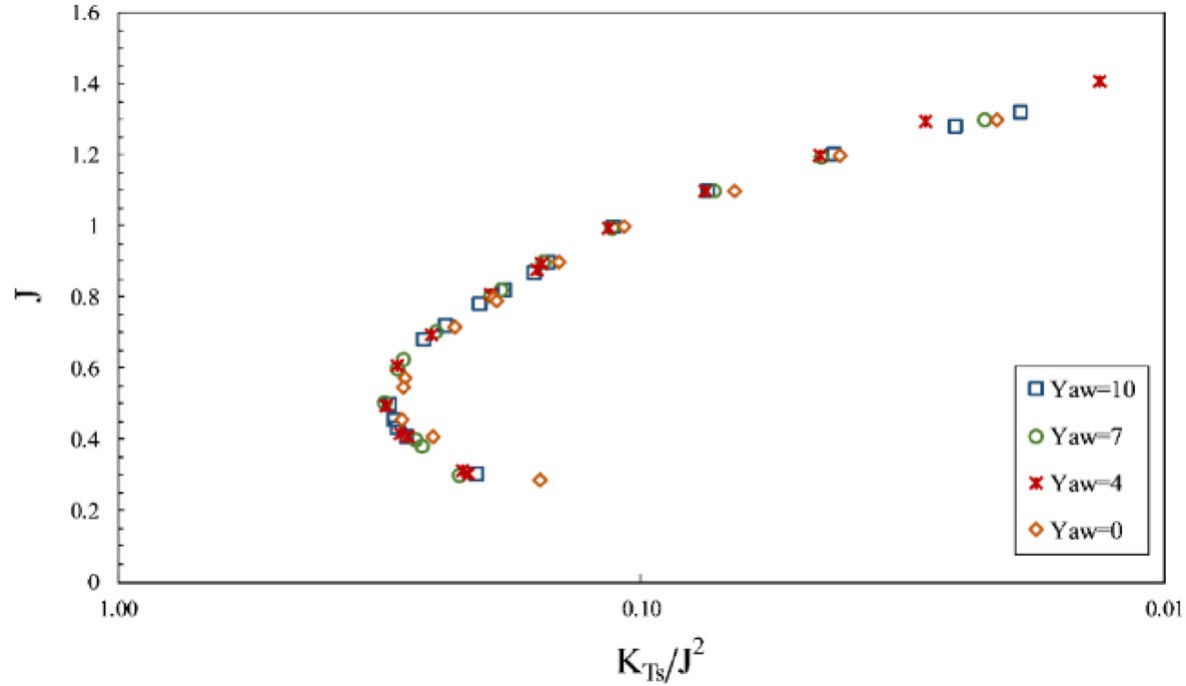
The effect of yaw angle on the thrust coefficient in the propulsion direction ($I=0.4$).

**Figure 29:**
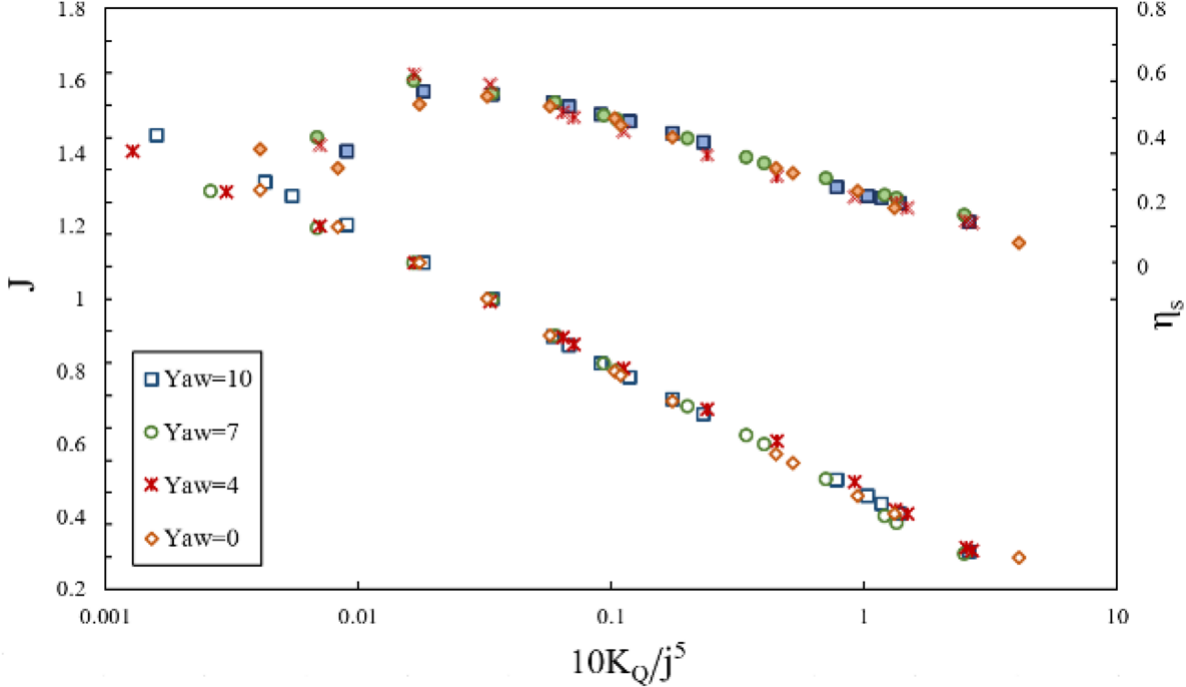
The effect of yaw angle on torque coefficient and efficiency in the propulsion direction ($I=0.6$).

Through calculating the thrust force in advance direction, the impact of the side force conversion in that direction could be observed for the propulsion thrust coefficient (*K*_*TS*_/*J*^2^) in Fig. 28. The collected data showed that increased yaw angle, in general, changed the propulsion thrust force. Yet, the level of thrust change for various advance ratios would be different regarding a combination of hydrodynamic parameters, blade ventilation, and the degree of change in the inclination angle for each section against the flow and does not have a fixed approach to the yaw angle in advance ratios. The yaw angle generated a higher impact at each immersion ratio under partial ventilation than the fully ventilation mode. Moreover, Fig. 29 showed slight changes in the torque coefficient, resulting from changes in propeller effective pitch and the inclination angle due to flow direction change against the propeller blades. The efficiency in line with the propulsion also changed with increased yaw angle from the yaw 0º, within a 5% range.

**Figure 30:**
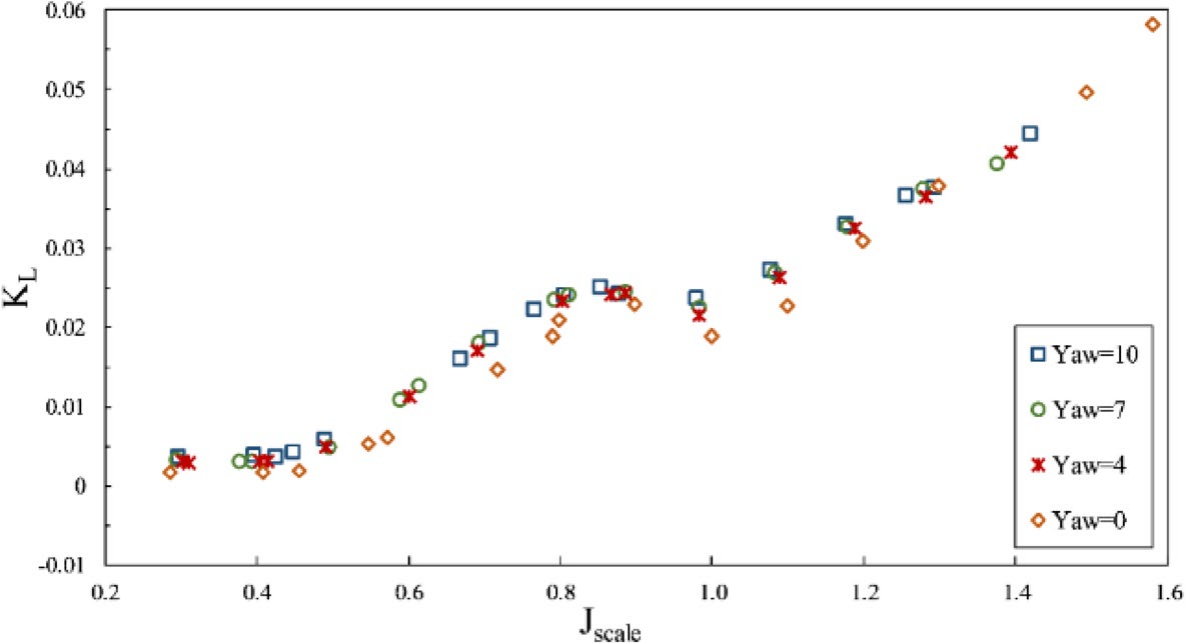
The effect of yaw angle on the lift coefficient in the propeller direction ($I=0.6$).

**Figure 31:**
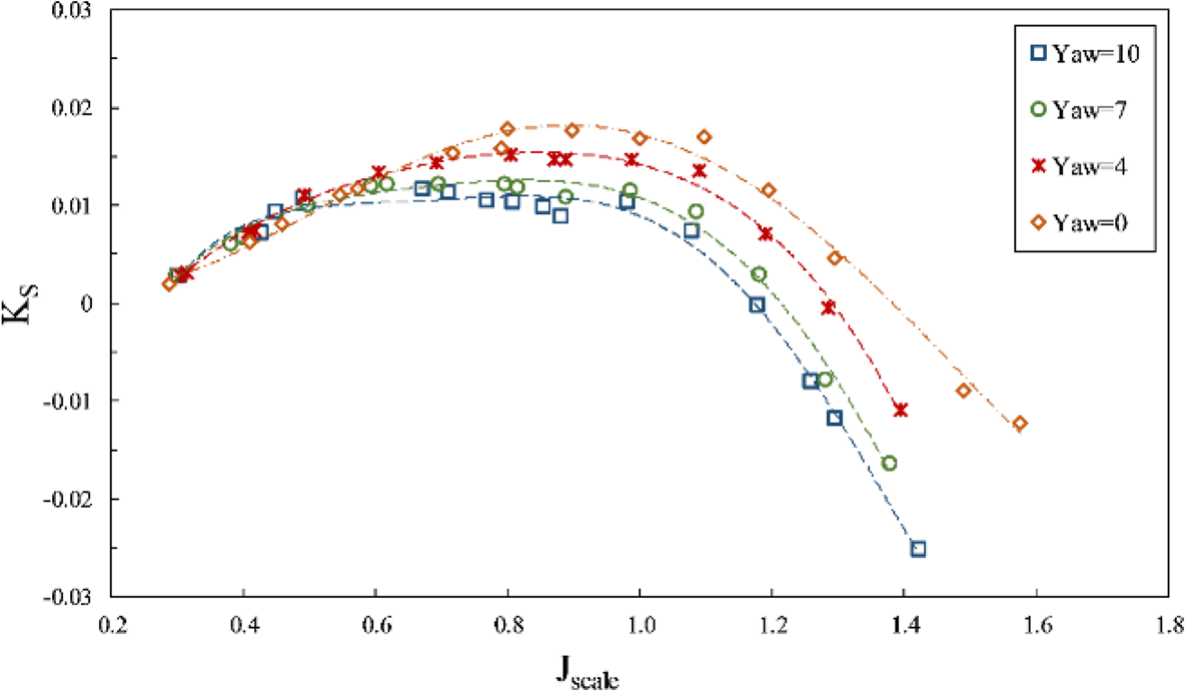
The effect of yaw angle on the side force coefficient in the propeller direction ($I=0.6$).

According to Fig. 30, the lift force on the shaft also grew with increased yaw angle, while Fig. 31 pointed to the reduction of the side force on the shaft due to reduced effective pitch of the propeller in line with the flow*.* Based on comparing the side force and thrust in line with the flow, it can be seen that with the increased yaw angle, the horizontal force was reduced significantly against the propulsion due to the reverse direction effect generated by the thrust force under the generated yaw angle. For instance, at an immersion ratio of 0.6, in the maximal conditions, the ratio of the side force to thrust ($\frac{{F}_{H}}{T}$) from 0.6 at the yaw angle 10º to 0.05. Such a change signified the side force conversion into thrust along the propulsion and slightly increased propulsion efficiency.

## Comparing results with estimated data from design phase

**Figure 32:**
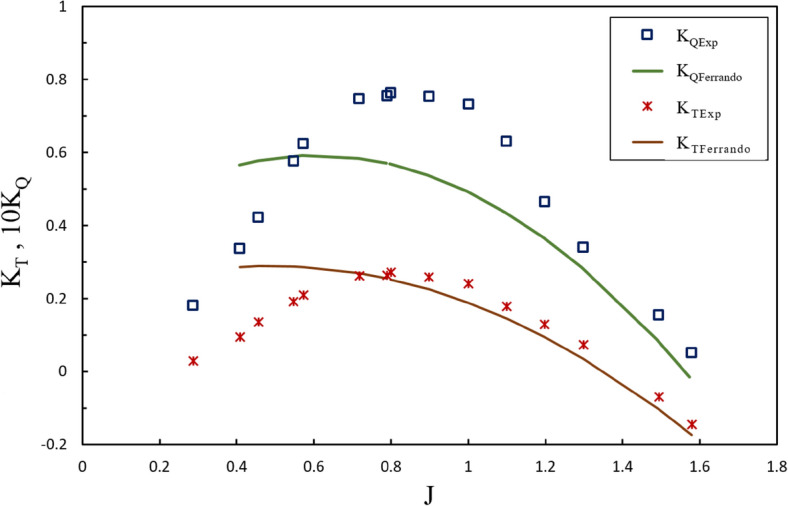
Comparison of Results with Initial Estimates of Design Phase ($I=0.4,\alpha =8^\circ$).

**Figure 33:**
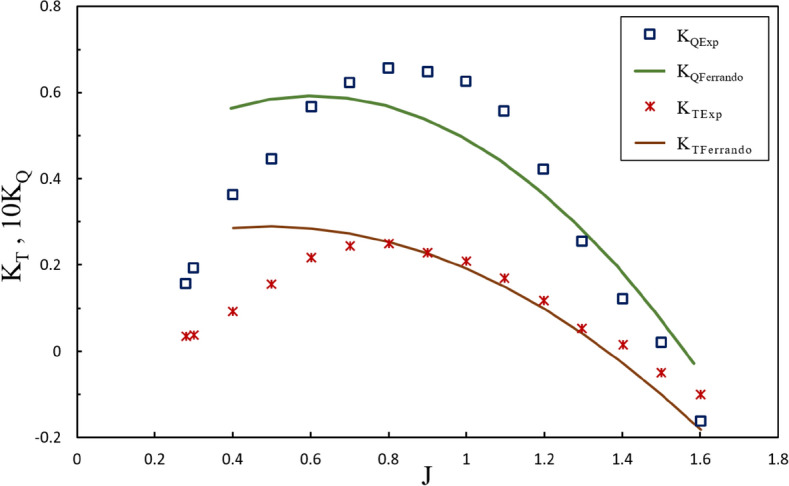
Comparison of Results with Initial Estimates of Design Phase ($I=0.6,\alpha =6^\circ$).

Estimating torque and thrust hydrodynamic coefficients has been challenged for the design phase of SPPs, and comprehensive relations have not been developed to project their performance. In order to assess the equations applied to the design phase of an HL002 propeller after the experimental tests, the hydrodynamic coefficients of the propeller at different positions were contrasted with Ferrando’s quadratic regression equations for four bladed propellers^2^, such as the hydrodynamic coefficients obtained at immersion ratios of 0.4 and 0.6 for two inclination angle (6º and 8º), as depicted in Figs. 32 and 33. According to experimental data, the regression thrust equation estimated the thrust coefficient with an error of less than 20% only under the partial ventilation region at $0.8\le J\le 1.5$, while the precision of such calculations diminished at increasing immersion ratios and inclination angle. Moreover, changes in the yaw angle did not significantly affect the output of the equations. Yet, the torque coefficient was not estimated with sufficient accuracy by the regression equations at any advance ratios of the design phase, and the minimum error occurred at 15% for the advance ratio of 1.3. Such performance points to insufficient accuracy of the equations and their failure to include all the parameters affecting the propeller performance. Considering the high operational costs of such propellers, the results highlight the need for experimental testing of the propeller after the design phase.

## Conclusion

In this research, the model test results of a 4-blade custom designed propeller were discussed, and its performance was compared with the design criteria. The present study considered the wake development manner of surface-piercing propellers at different advance ratios, the Froude number's impact, and the independence ranges proposed by the previous studies. According to the extracted experimental results, the independence ranges suggested by Olofsson were selected as suitable for model testing SPPs. Moreover, the model propeller performance curve was drawn for different test conditions, and the propeller behavior was identified according to different positional parameters.

In the design algorithms used for this propeller, Ferrando’s quadratic regression equations for four-bladed propellers were employed to estimate thrust and torque coefficients. Comparing the experimental data obtained and the estimations of the design phase, it can be observed that the equations estimated the thrust coefficients with an error of less than 20% only under the partial ventilation region at $0.8\le J\le 1.4$. In comparison, the torque coefficients were experienced an error above 30% within the same range. This will lead to an inaccurate estimate of the power required to operate the propeller, as well as an inappropriate selection of engine, while the propeller performance will be estimated with an error at least 25%. Comparison of the present research results with those by Lorio or Seyyedi et al. on the amounts estimated by Ferrando’s equations shows the different behaviors of these equations for various geometries. Such observations pointed to the incomplete coverage of impacts by the geometrical parameters on the propeller, such as blade section shape and type of radial distribution, like rake, skew and pitch ratio of the propeller in the regression equations. This shortcoming of the equations can be attributed to the insufficient experimental data about the different geometries of propellers.

The maximum thrust generated by a propeller was identified as one of the most important and effective parameters in designing propellers and propulsion systems. A propeller with optimum efficiency is desirable to generate the required thrust for starting conditions with low advance ratios, velocity increase and vessel planning mode. The present study thus addressed the impact of different position parameters on propeller efficiency and thrust.

The experimental results pointed to the favorable impact of increased immersion ratio on promoting propeller thrust; also, propeller efficiency has the highest value in the immersion ratio range of $0.4 <\mathrm{I }<0.75$ at $\mathrm{J }= 1$, but does not have good efficiency in lower advance ratios and immersion depth and its lower than the design estimate. The results show that increasing the inclination angle in all cases will not increase thrust and performance. The optimum inclination angle for increased thrust and efficiency in line with the propulsion is 6 degrees.

Changing the propeller yaw angle will theoretically strengthen the aggregate thrust toward the propulsion and increase efficiency while minimizing the side force. However, the results generally do not indicate a significant increase in propulsion efficiency and thrust for higher yaw angles up to 10°. However, at advance ratios smaller than ${j}_{cr}$, it will increase thrust. These results show different behavior depending on the geometry.

Another interesting point was the zero-load point on the propeller performance curve, reported approximately at range of 1.4 by the information from the thrust coefficient figures, while the zero-load point is theoretically determined in accordance with propeller pitch ratio at $\frac{P}{D}=1.24$. Such difference in effective propeller pitch can be attributed to the blade geometry's impact, such as the impact of the cup on the high-pressure face and thus reveals the considerable impact of blade geometry on its performance.

Furthermore, the information pertaining to propeller lateral forces was considered under different operational and position conditions, which could help identify propeller behavior, determine and compare the impacts of different geometries, and design shafts, supports, and bearings as required. By comparing the results of this study with experimental studies performed on SPP’s differential geometry, the effect of blade geometry on propeller behavior under different positional parameters can be observed. Finally, the present study's information will be used to develop a numerical solution method for surface-piercing propellers and optimize the propeller geometry to generate higher thrust.

## Data Availability

The datasets generated during and/or analyzed during the current study are available from the corresponding author on reasonable request.
